# Admixtures for concrete and mortar with MgO-based binders: a review from RILEM TC 311-MBC

**DOI:** 10.1617/s11527-026-03345-5

**Published:** 2026-11-04

**Authors:** Matthias Müller, Sanket Rawat, Hoang Nguyen, Clélia Bouchaud-Deliot, Raphael Kuhn, Dan Meng, Pauline Quiatchon, Jishen Qiu, Navid Ranjbar, Zeyu Zhou, Frank Winnefeld

**Affiliations:** 1 F.A. Finger-Institut für Baustoffkunde, Bauhaus-Universität Weimar, Coudraystrasse 11, 99423 Weimar, Germany; 2 School of Civil and Environmental Engineering, University of Technology Sydney (UTS), Sydney, NSW 2007 Australia; 3 Fibre and Particle Engineering Research Unit, University of Oulu, Pentti Kaiteran Katu 1, 90014 Oulu, Finland; 4 Swiss Federal Laboratories for Materials Science and Technology, Laboratory for Concrete and Asphalt, Überlandstrasse 129, 8600 EmpaDübendorf, Switzerland; 5 Institute of Geological Sciences, Mineralogy Group, University of Bern, 3012 Bern, Switzerland; 6 Swiss Federal Institute of Technology, Institute for Building Materials (IfB), ETH Zurich, 8093 Zurich, Switzerland; 7 Department of Civil and Mechanical Engineering, Technical University of Denmark, 2800 Kongens Lyngby, Denmark; 8 Department of Civil and Environmental Engineering, The Hong Kong University of Science and Technology, Clear Water Bay, Kowloon, Hong Kong China

**Keywords:** Magnesia-based cements, Admixtures, Plasticizer, Hardening accelerator, Retarder, Alkali phosphates

## Abstract

Magnesia-based cements are an emerging, potential low-CO_2_ alternative to Portland cements. Admixtures, which control workability, setting and hardening are crucial for the application of these novel binders in mortar and concrete mixtures. However, research and development in this field are not yet well advanced. Working Group 4 of RILEM TC 311-MBC 'Magnesia-based binders in concrete' critically reviewed recent advances and further perspectives in the field of admixtures for magnesium silicate and magnesium carbonate cements and concretes, focused mainly on plasticizers, accelerators and retarders. Alkali phosphates are very efficient plasticizers, especially for magnesium silicate cements. Also, polycarboxylate ethers work, when rather high dosages are applied, despite that current products are designed for Portland cements. Furthermore, alkali phosphates act both as hardening accelerators and retarders, depending on the type of magnesia-based cements. Carbonates, silicates and organic salts, i.e. magnesium acetate, have been used as hardening accelerators, while certain metal cations and organic anions such as citrate and gluconate have been used as retarders. However, further research is needed to develop admixtures specifically for these two groups of magnesia-based cements which facilitate good workability and strength development.

## Introduction

In recent years, magnesia-based cements have experienced a renaissance as a possible low-carbon alternative to Portland cement (PC)-based binders [1–3] Though they have been established for a long time, their use is still limited to niche applications [4]. This can be attributed to the specific properties of these cements, which comprise a wide range of different chemistries including magnesium oxychloride, oxysulphate, phosphate, carbonate and silicate [1].

Hydrated magnesium oxychloride cements (so-called Sorel cements) and magnesium oxysulphate cements are not stable in moist environments [5], which has prevented their widespread use in concrete. Examples of applications include floors or fire-resistant panels [4]. Magnesium phosphate cements, on the other hand, have the potential to be used in load-bearing structural elements due to their good mechanical performance and durability. However, since phosphates are of paramount importance to the fertilizer industry, the mass use of magnesium phosphate cements would not be sustainable and they are primarily used as rapid setting and hardening cements for repair applications [1].

In contrast, magnesium silicate and magnesium carbonate cements have the potential to be used as a mass product in the construction industry as a low-carbon binder for concrete or mortar, provided that the production of MgO does not result in high CO_2_-emissions. Currently, MgO is mainly sourced by the calcination of magnesite [4], which is not sustainable from a CO_2_ perspective. The prerequisite for a low CO_2_ footprint is the extraction of reactive MgO from seawater desalination brines or MgO and SiO_2_ from carbonate-free Mg-based minerals [1, 6, 7]. Several approaches to the latter are currently being developed [8, 9]. In view of their particular relevance for concrete applications, the present review covers only the use of admixtures for magnesium silicate cements and magnesium carbonate cements.

Magnesium silicate cements contain sources of reactive MgO and silica (e.g. silica fume). Their hydration follows a sequential process, where firstly brucite (Mg(OH)_2_) is formed, which results in a slightly alkaline pore solution (e.g. with a pH value of 10.5 [10]). Brucite subsequently reacts with silica to form magnesium silicate hydrate (M-S-H) as strength giving phase [11–13]. M-S-H formation is a slow process, as brucite must provide the dissolved Mg^2+^ and OH^−^ ions to react with the silica, but the solubility of brucite at pH levels above 10.5 is very low [14]. In addition to pure silicates, aluminosilicates (e.g. metakaolin) can also be used with MgO, leading to the formation of magnesium aluminosilicate hydrate (M-A-S-H) and hydrotalcite-like phases [15].

Magnesium carbonate (MC) cements consist of blends of reactive MgO and hydrated magnesium carbonates (HMCs) such as hydromagnesite (Mg_5_(CO_3_)_4_(OH)_2_ · 4H_2_O) and nesquehonite (MgCO_3_ · 3H_2_O) [2, 3, 16–19]. Upon hydration, hydrous carbonate-containing brucite (HCB), a brucite-like phase of low crystallinity with stacking faults due to the presence of water and (bi)carbonate in the interlayer, is formed, resulting in high early strength [18–20]. Much of the literature related to the application of admixtures for magnesium carbonate cements covered in the present review focuses rather on pure magnesium oxide to make carbonatable binders or used as a model substance in basic research on admixtures. Much less information is available on the use of admixtures for magnesium carbonate cements. It should be noted that the findings regarding the interactions between admixtures and MgO are only applicable to magnesium carbonate cements to a certain extent, and that further research is needed to substantiate these findings. Comprehensive overviews on the different magnesia-based binders are given in [1–4].

Magnesia-based binders may only find widespread application, if they can be used to produce concrete for building components and structures. A key property of concrete is its plasticity in fresh state, when it can be placed in formwork, where it then hardens through the chemical reaction of the binder with water. To achieve this, concrete must have sufficient workability (i.e. low yield stress and low plastic viscosity) in an adequate timeframe for placement and compaction, followed by rapid development of its microstructure and strength. The challenge for magnesia-based binders is to replicate these properties to a similar extent as for PC-based binders.

Magnesia-based binders generally require more water than PC to get a workable mix. This is mainly due to the high specific surface area of the starting materials (e.g. reactive MgO, silica fume) [21]. Therefore, plasticizers are crucial for reducing the water demand, in order to achieve a dense microstructure and good strength of the hardened material. While plasticizers used for PC-based binders can serve this purpose, they require significantly higher dosages [22]. Although alternative effective dispersing agents based on alkali phosphates have been found for magnesia-based binders [23], the production of concretes with a sufficiently low water-cement ratio remains challenging.

Furthermore, the hardening process of magnesium silicate cements is often slow when compared to most calcium-based binders. Effective hardening accelerators are required to achieve a sufficient hydration degree within a practical period of time [24]. On the contrary, fast setting can occur with magnesia-based binders, especially in the case of magnesium carbonate cements, which makes it necessary to use retarders to ensure a sufficient timeframe for placing [25]. Other admixtures such as air-entraining agents or shrinkage reducers are also required for certain concrete applications. Research in this area is still in its infancy. However, a few examples show that admixtures known from PC systems may also be effective here.

Generally, the use of magnesia-based binders in concrete, even at the laboratory scale, is still in early stages despite some recent work [25–29], as the production of workable concrete with good strength and durability remains a significant challenge. Addressing this satisfactorily will be a basic prerequisite for these binders to establish themselves as an alternative to Portland cement. Effective admixtures will play a key role to achieve this goal. Accordingly, the present review is intended to provide an overview of the current state of research on admixtures for concretes with magnesium silicate and magnesium carbonate cements and to identify future research priorities.

## Plasticizers

Magnesium silicate and magnesium carbonate cements have a high water demand. Plasticizing admixtures are therefore essential to ensure a good workability at acceptable water-cement ratio. Predominantly alkali phosphates and polycarboxylatether (PCE)-based plasticizers have been used for that purpose.

### Alkali phosphates

#### General information on alkali phosphates

Alkali phosphate-based dispersants have been frequently used to improve the workability of magnesium silicate cements [10, 13, 22–24, 30–35] and of pure MgO-binders [36–42], with sodium hexametaphosphate ((NaPO_3_)_6_, NaHMP^1^) as the most common admixture.

Alkali phosphates are salts of phosphoric acid (H_3_PO_4_). They contain PO_4_-tetrahedra (orthophosphate) as characteristic structural element, which can be linked via common oxygen atoms to produce polymers with a chain, cyclic or cross-linked structure. Alkali phosphates can be structurally distinguished according to their degree of polymerisation and their oxide ratios (Na_2_O, K_2_O, H_2_O to P_2_O_5_) [43].

Monophosphates have isolated PO_4_-tetrahedra and an oxide ratio of 3. The term polyphosphate describes phosphate salts with a chain structure. They have an oxide ratio of > 1 to 2. The polyphosphate group contains oligomeric phosphates with a short chain length, but also high-molecular polyphosphates. Phosphates with chain length 2 are termed diphosphate (pyrophosphate), with a chain-length of 3 they are termed triphosphate (tripolyphosphate), etc. [43].

Phosphates with a cyclic structure are called metaphosphates. They have an oxide ratio of 1. As high-molecular polyphosphates almost have an oxide ratio of 1, they are often confused with metaphosphates. A typical example is the frequent use of the term sodium hexametaphosphate for commercial products that mostly contain high-molecular, straight-chain polyphosphates [43]. This makes it considerably more difficult to assess the effectiveness of alkali phosphates, as sodium hexametaphosphate is the most commonly used alkali phosphate for magnesia-based binders. It is therefore recommended that studies using alkali phosphates as admixture include a comprehensive characterization. Relevant parameters include chain length distribution, molecular structure, purity and storage age.

In aqueous solutions condensed phosphates, such as poly- or metaphosphates, decompose by hydrolysis into smaller structural elements [44]. The rate of hydrolytic degradation depends not only on the structure of the polyphosphate, but also on various factors including temperature and pH of the solution [45]. Dissolved metal cations can increase the rate of hydrolysis in alkaline conditions [46]. Cross-linked phosphates are not commonly used commercially [43].

#### Dispersing mechanism of alkali phosphates

**Fig. 1:**
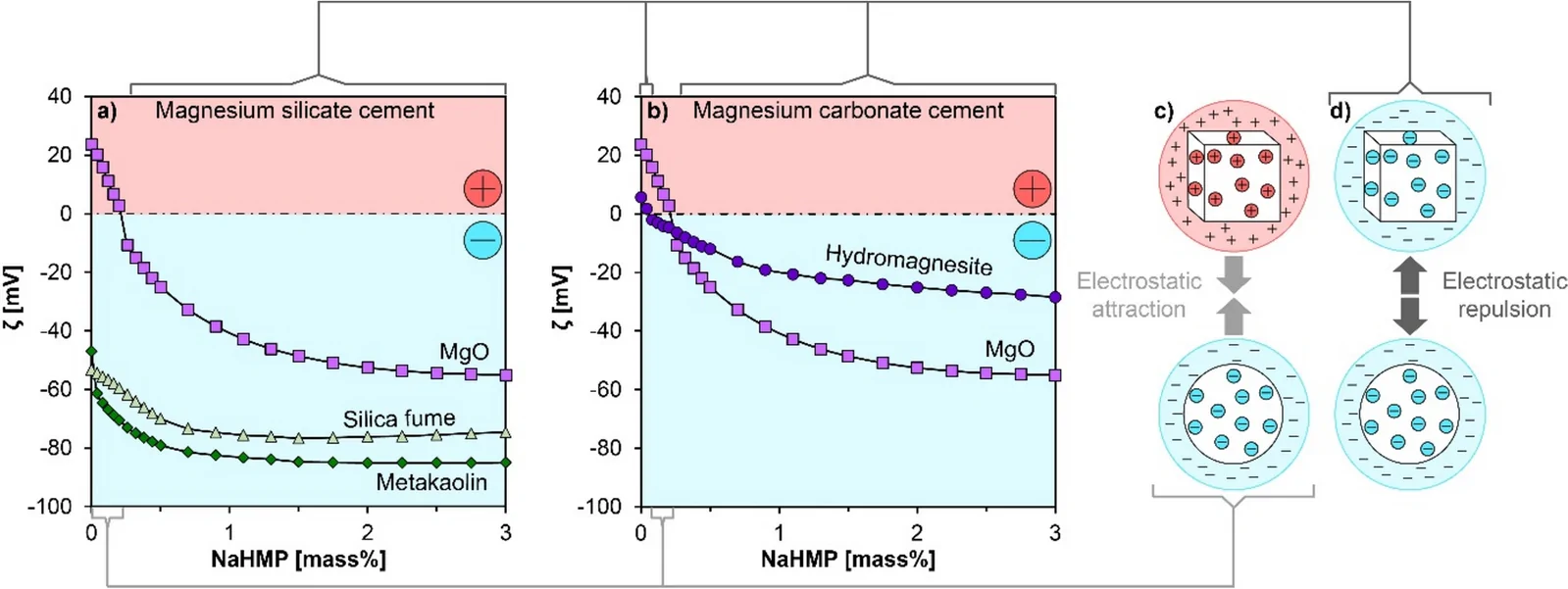
Illustration of the transition from electrostatic attraction to electrostatic repulsion as a function of NaHMP concentration in **a** magnesium silicate systems and **b** magnesium carbonate systems, reflected by changes in the zeta potential of hydromagnesite, MgO, silica fume, and metakaolin (adapted from [47]). Red regions indicate a positive zeta potential, whereas blue regions indicate a negative zeta potential. Regions dominated by **c** electrostatic attraction between oppositely charged particles are highlighted in light grey below the plots, while regions dominated by **d** electrostatic repulsion between similarly charged particles are highlighted in dark grey above the plots. (colour figure online)

A recent study proposed a dispersion mechanism for alkali phosphate admixtures with magnesia-based cements [47]. Zeta potential measurements of representative raw materials for magnesium silicate cements (MgO, silica fume and metakaolin) and magnesium carbonate cements (MgO and hydromagnesite) with increasing NaHMP dosages revealed a systematic shift toward more negative surface charges (Fig. 1).

For magnesium silicate cements, increasing NaHMP concentrations progressively neutralize positive surface charges (e.g. on MgO), thereby suppressing electrostatic attraction between oppositely charged particles (e.g. MgO and metakaolin/silica fume). At concentrations above approximately 0.2 mass-% (with respect to dry binder mass), the overall negative surface charge dominates, leading to enhanced electrostatic repulsion.

For magnesium carbonate cements the situation can be more complex, as hydromagnesite and MgO particles both carry positive surface charges, when no NaHMP is present in the system [47]. Consequently, without NaHMP electrostatic repulsion occurs. With increasing NaHMP concentration, the changes in the surface charges reduce the electrostatic repulsion, eventually causing electrostatic attraction in the concentration range, where hydromagnesite and MgO carry opposite charges. At NaHMP concentrations exceeding 0.4 mass-% (with respect to dry binder mass), the absolute surface charge of hydromagnesite and MgO particles exceeds the initial values, resulting in stronger repulsive forces than in magnesium carbonate systems without NaHMP.

The mechanism of action of NaHMP in these systems is described in detail in [47]. Upon addition, the polyphosphate species deprotonate under the highly alkaline conditions of the cement system. These negatively charged species adsorb onto positively charged particle surfaces, forming surface-bound linear polyphosphate complexes, as demonstrated by ^31^P nuclear magnetic resonance spectroscopy. This adsorption process imparts an increasingly negative surface charge to the particles. The resulting shift from a positive to an increasingly negative surface charge explains the observed changes in zeta potential and the transition from electrostatic attraction to electrostatic repulsion. Consequently, the reduced interparticle attraction and increased electrostatic repulsion lower the stress required to initiate flow, thereby improving the rheological performance and explaining the effectiveness of NaHMP as a plasticizer [47].

#### Effect of alkali phosphates on the workability of magnesium silicate cements

Early applications of NaHMP in magnesium silicate systems can be found for refractory castables based on dead-burned MgO and silica fume, e.g. in [48]. Contrary to the reactive MgO used in magnesium-silicate and magnesium carbonate cements, dead-burned MgO has a lower reactivity and a lower specific surface area, due to its calcination at temperatures above 1200 °C [49]. The use of NaHMP as an admixture for magnesium silicate cements was first reported in [24], where it was found to be a very effective water reducer for the preparation of pastes based on MgO and silica fume.

Generally, the most effective concentration range for NaHMP was identified as 1–2 mass-% referred to total binder [13, 30]. Doses exceeding a certain threshold (e.g. 5 mass-% [13]) resulted in a significant loss of fluidity, as indicated by the decreased spread in mini-slump tests The mechanism underlying the observed upper thresholds in magnesium silicate cement systems has yet to be elucidated.

Sodium trimetaphosphate ((NaPO_3_)_3_, NaTMP), sodium orthophosphate (Na_3_PO_4_, NaOP), and dipotassium hydrogen phosphate (K_2_HPO_4_, DKP) were also found to be effective dispersants for magnesium silicate cements [31, 33, 35].

**Fig. 2:**
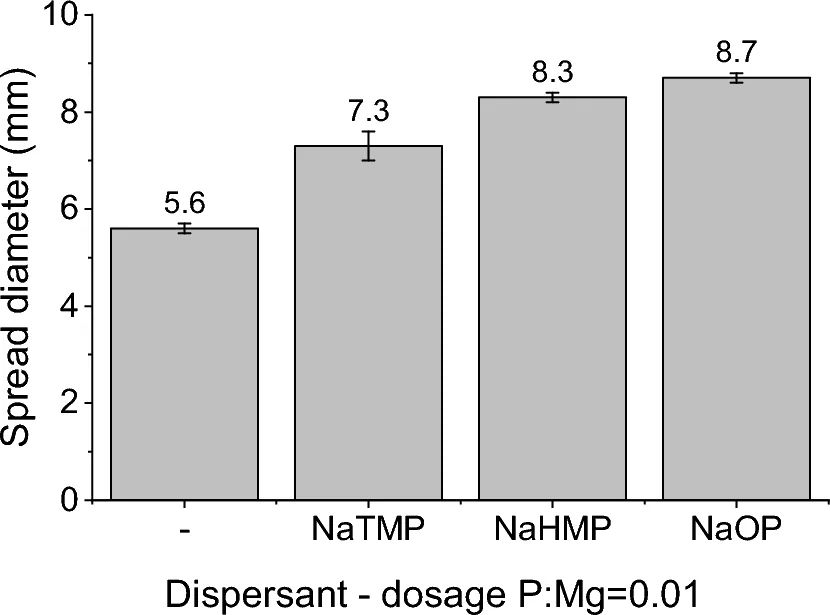
Effectiveness of different alkali phosphates on workability of magnesium silicate cement at w/b = 2; based on data from [33]; flow test carried out with a mini slump cone (height 57 mm, upper diameter 19 mm, bottom diameter 38 mm) placed on a glass plate

The effectiveness of NaHMP, NaTMP, and NaOP was compared in various studies with different results [31, 33, 50]. In [33], the effectivity of these admixtures on magnesium silicate cements (MgO and SiO_2_ in a 1:1 molar ratio) was compared by using mini-slump tests. The dosage of the dispersants was determined to correspond to a P:Mg ratio of 0.01, which is equivalent to 1.0 mass-% for NaHMP and NaTMP, and 1.6 mass-% for NaOP. Their study showed a workability improvement in the order of NaTMP < NaHMP < NaOP (Fig. 2), which was linked to the increasing concentration of orthophosphate ions (PO_4_^3−^) in solution in the same order.

Using similar alkali phosphates at a concentration of 1 mass-%, [31] reported workability results for a MgO-SiO_2_-system (1:1 by mass) with w/b = 0.4. using the ASTM C1437-07 flow table test. Workability improved in the order of NaOP < NaHMP < NaTMP. These alkali phosphates were also studied with rheological measurements (concentric cylinder geometry) in [50] at a dosage of 2 mass-% in a MgO–SiO_2_-system (1:1 by mass) with w/b = 0.55. There, NaHMP was the most effective plasticizing admixture, which resulted in the lowest static and dynamic yield stress and thixotropy, followed by NaTMP and NaOP.

Beside variations in the binder composition and the applied alkali phosphate dosages, the notable differences in the performance of the various admixtures in the abovementioned studies [31, 33, 50] may also be related to the assessed workability and rheological parameters and/or the mixing procedure. In [33], the admixtures (powders) were dry mixed with the binder, whereas in [31, 50], they were dissolved in the water before adding the binder, thus ensuring a more immediate effect and initiating hydrolysis of the condensed alkali phosphates. Dissolving alkali phosphate powder in the mixing water was the standard procedure in most studies [6, 10, 12, 13, 23, 30, 32, 34, 35, 50–61], though some exceptions exist [33, 62–64] or the mixing procedure was not clearly stated in some cases [24, 65, 66]. Whether the mixing procedure has a significant effect on the workability and performance of magnesium silicate cements has yet to be evaluated. A delayed addition might have an advantage as well, as for PCE in the case of Portland cement [67]. It should also be noted that direct comparisons between studies on alkali phosphates are complicated by differences among the individual products and by the limited shelf life, which can also cause changes in composition (e.g. chain length). Furthermore, commercial metaphosphates sometimes contain high-molecular polyphosphates instead of metaphosphate [43].

**Fig. 3:**
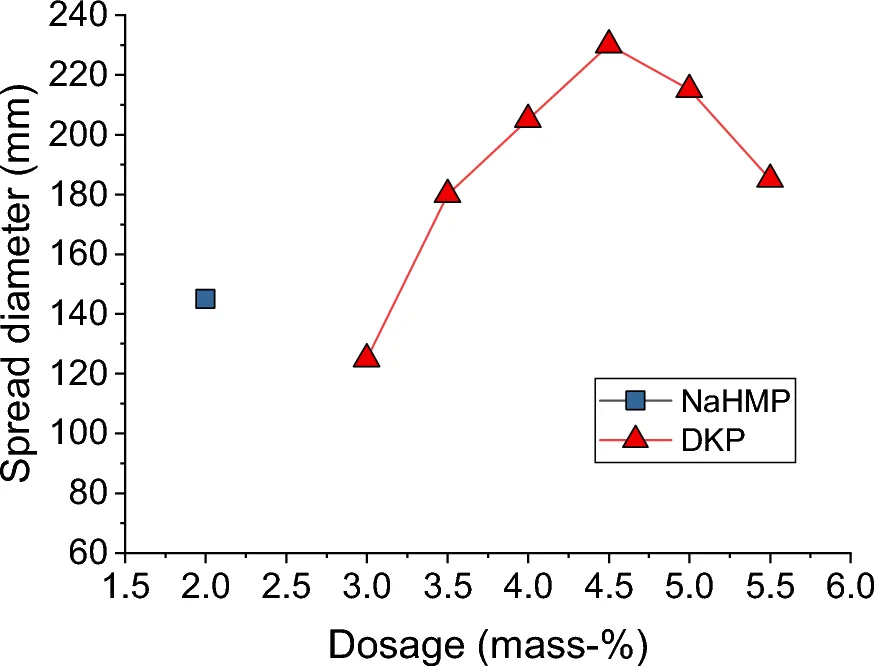
Effectiveness of DKP and NaHMP on workability of magnesium silicate cement at w/b = 0.45; based on data from [35]; flow test carried out with a mini slump cone (height 60 mm, upper diameter 36 mm, bottom diameter 60 mm) placed on a glass plate

In [35], the plasticizing potential of DKP and NaHMP was compared in a MgO/silica fume (40:60) system with w/b = 0.45. In a direct comparison at a dosage of 2 mass-%, DKP was less effective than NaHMP, not yielding a workable paste. However, the effectivity threshold of DKP was observed at 4.5 mass-%, which is notably higher than the typical effective threshold dosage (1–2 mass-%) reported for NaHMP (e.g. in [13, 30]). With 4.5 mass-% DKP, a higher fluidity (mini slump spread diameter, Fig. 3) and lower dynamic yield stress and plastic viscosity (rheometer measurements with concentric cylinder geometry) than with 2 mass-% NaHMP occurred. Unfortunately, the significance of the results presented is very limited, as only a NaHMP dosage of 2 mass-% was tested in the system under consideration.

#### Effect of alkali phosphates on the workability of magnesium carbonate cements

**Fig. 4:**
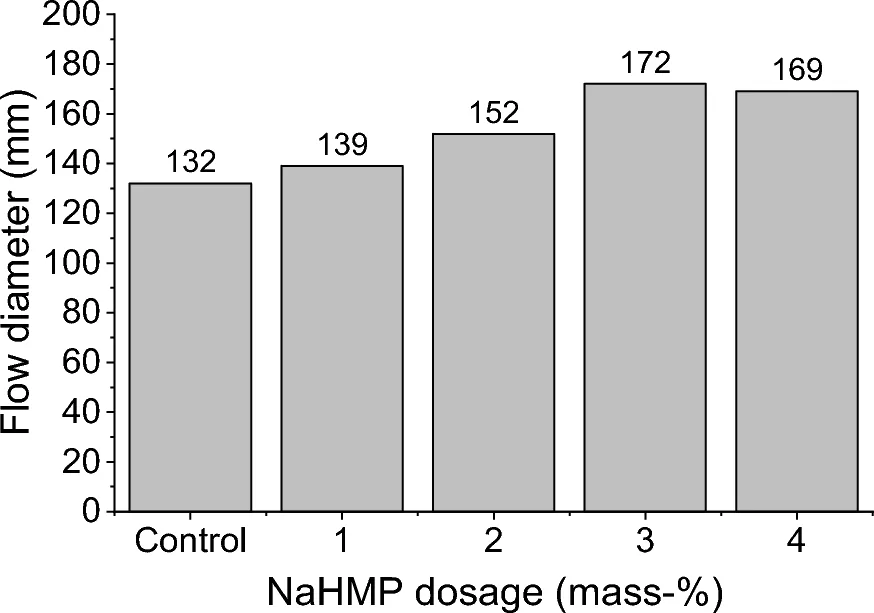
Flow spread of magnesia-based paste with varying amounts of NaHMP in mass-% (cone dimensions (height 50 mm, upper diameter 70 mm, bottom diameter 100 mm), adapted from [68]

While the use of NaHMP in reactive MgO systems is relatively well-documented [36–42, 68], no systematic studies for NaHMP or other alkali phosphates as plasticizers for magnesium carbonate cement systems exist. In [68], the influence of NaHMP dosage on the consistency of reactive MgO pastes (w/b = 0.7) was evaluated using the flow table test (following ASTM C230 (2023)) across a range of 0–4 mass-%. The results demonstrated an increase in flow spread with increasing NaHMP content up to a dosage of 3 mass-%. A slight reduction in flowability was observed beyond this dosage, suggesting the presence of an optimal threshold (Fig. 4). The occurrence of such a threshold has also been reported for magnesium silicate cements (e.g. in [13, 23, 30]). In [68], NaHMP also retarded the setting of the reactive MgO-cement, which may be beneficial for ensuring a sufficient workability time.

### PCE-based plasticizers

#### Dispersing mechanism of PCE-based plasticizers

For magnesia-based cement composites, only little is known on the mechanism of PCE, though there are some studies showing the effect on individual raw materials as MgO and silica fume. As surface chemistry and adsorption properties of MgO towards PCE are similar to those of Portland cement [69], dead-burnt MgO has been used as non-reactive model material for Portland cements surfaces in atomic force microscopy, zeta potential, rheology and PCE adsorption experiments [70–73].

In dead burnt MgO systems, PCE molecules strongly adsorb with their anionic backbones (commonly negatively charged carboxylate groups (-COO⁻)) onto the positively charged MgO surfaces [72]. With this adsorption, the zeta potential changes from positive to zero or even negative. This might potentially reduce the electrostatic attractions with negatively charged particles, e.g. silica fume. While the negative backbone is adsorbed at the solid–liquid interface of MgO, the uncharged side chains (usually hydrated polyethylene glycol (PEG) side chains) face the liquid and impart repulsive inter-particle forces of steric origin [71]. These electro-steric interactions reduce or eliminate the adhesion between particles. With increasing length of the ethylene oxide side chains from the adsorbed PCE, the degree of flocculation in the MgO system is reduced [71]. Based on these results obtained with dead-burned MgO, it can be expected that PCE-based plasticizers are also effective with reactive MgO, the principal constituent of magnesium silicate and magnesium carbonate cements.

In [69], MgO and Mg(OH)_2_ were compared as model powders to study admixture interactions. This comparison could be particularly relevant for understanding the interaction of admixtures with reactive MgO, which undergoes hydration to form Mg(OH)_2_. Both MgO and Mg(OH)_2_ showed distinct behaviours in terms of polymer adsorption (depending on the type of polymer) and surface charge characteristics. One of the main factors responsible for the differences in adsorption behaviour appears to be the pH. Although both MgO and Mg(OH)_2_ possess positively charged surfaces at their respective pH values of approximately 12 and 11.3, the higher OH^−^ concentration at the higher pH is proposed to compete with PCE molecules for adsorption sites.

Tayebi et al. [74] studied seven different PCE-based superplasticizers in reactive MgO cement at dosages ranging from 0.2 to 1.0 mass-% of the dry binder. Rheological measurements indicated a shear-thinning flow behaviour, and a consistent reduction in both yield stress and plastic viscosity with increasing PCE dosage was found. Thixotropy also decreased, likely due to increased particle spacing and modified colloidal interactions. In addition to rheological modifications, the PCE also exhibited a complex effect on the hydration kinetics. Initially hydration was retarded for up to 60 min. Nonetheless, the initial setting was accelerated whereas the final setting was delayed. In combination with calorimetry measurements, which showed a higher cumulative heat release after 72 h for mixes with PCE, this was interpreted as an extension of the hydration process. At the age of 28 days, no notable effect of the PCEs on phase assemblage and microstructure of the MgO pastes was found.

Silica fume is widely used as a reactive SiO_2_ source in magnesium silicate cements. Insights into the interaction between silica fume and PCE superplasticizers can be drawn from studies investigating silica fume as a supplementary cementitious material in Portland cement systems. Due to the very fine nature of silica fume, its high specific surface area leads to a significantly increased water demand for achieving workable paste consistencies [75]. This high water requirement contributes to the generally lower workability observed in magnesium silicate cements compared to Portland cement. While silica fume is typically used in minor amounts to partially replace clinker in Portland cement [75], it is often a major constituent in magnesium silicate systems, amplifying its impact on rheological behaviour [22]. PCEs act primarily through steric hindrance [76, 77], with their adsorption onto silica fume particles driven by hydrogen bonding interactions with the polymer side chains [78]. The negatively charged backbone of the PCE repels the likewise negatively charged silica fume surface, limiting adsorption through electrostatic interactions. In contrast, the polyethylene glycol side chains are non-ionic and do not experience this repulsion. As a result, the adsorption affinity of PCE on silica fume is governed mainly by two key parameters (i) the length of the side chains and (ii) the carboxylic equivalent of the polymer. According to [78], an optimal PCE structure for use with silica fume should exhibit a high anionic charge density, i.e. high carboxylic equivalent and relatively short side chains (less than 3000 Da).

#### Effect of PCE on the workability magnesium silicate cements

For magnesium silicate cements, PCEs were frequently employed to enhance the workability, although detailed rheological characterization has often not been reported. A direct comparison between a PCE superplasticizer (solid content 36 mass-%) and NaHMP in a MgO/silica fume (40:60) mixture was conducted by [22]. The effectiveness of each admixture was evaluated based on the water content to achieve a certain workability (spread of 150 mm ± 5 mm, as per ASTM C1437-07). For the materials used in that study, the same amount of water required for the 1 mass-% NaHMP reference system was achieved with 1–2 mass-% of liquid PCE, i.e. at a similar content of the active component when compared to NaHMP. Notably, the greatest reduction in water demand while maintaining equivalent workability was observed at liquid PCE dosages of around 3–4 mass-%, which equals about 1–1.4% solids. Concentrations above 4 mass-% of the liquid product resulted in increased water demand to maintain the workability.

PCE superplasticizers have also been used in several other studies including concrete scale [15, 27–29, 79–83], though without a direct comparison of effectiveness with alkali phosphate-based dispersants. In general, for achieving adequate workability, the reported PCE dosage for the PCE with magnesium silicate cements was always notably higher than for corresponding Portland cement-based systems (e.g. 3 mass-% for MgO/silica fume (1:1 by mass)-mortar compared to 1 mass-% for Portland cement-based mortar in [79]).

#### Effect of PCE on the workability of magnesium carbonate cements

PCE-based superplasticizers have been utilized in magnesium carbonate cement paste/mortar [17, 19, 84] and in one instance in concrete [25, 26] to improve workability. The dosages used ranged from 0.15 to 4 mass-% of the liquid PCE. Selecting the optimum dosage is critical, as excessive PCE can have adverse effects on the properties of fresh mortar or concrete. As commonly observed for Portland cement based binders (e.g. in [85]), factors such as plasticizer chemistry, binder composition, and water-binder ratio strongly influence this dosage threshold. This was clearly demonstrated in flow table tests conducted on magnesia-based mortars using two commercial PCE-based superplasticizers [86], where doubling the dosage (from 2 to 4 mass-% of the dry binder) improved workability in case of one PCE, while for the other PCE, strong segregation occurred due to overdosage.

**Fig. 5:**
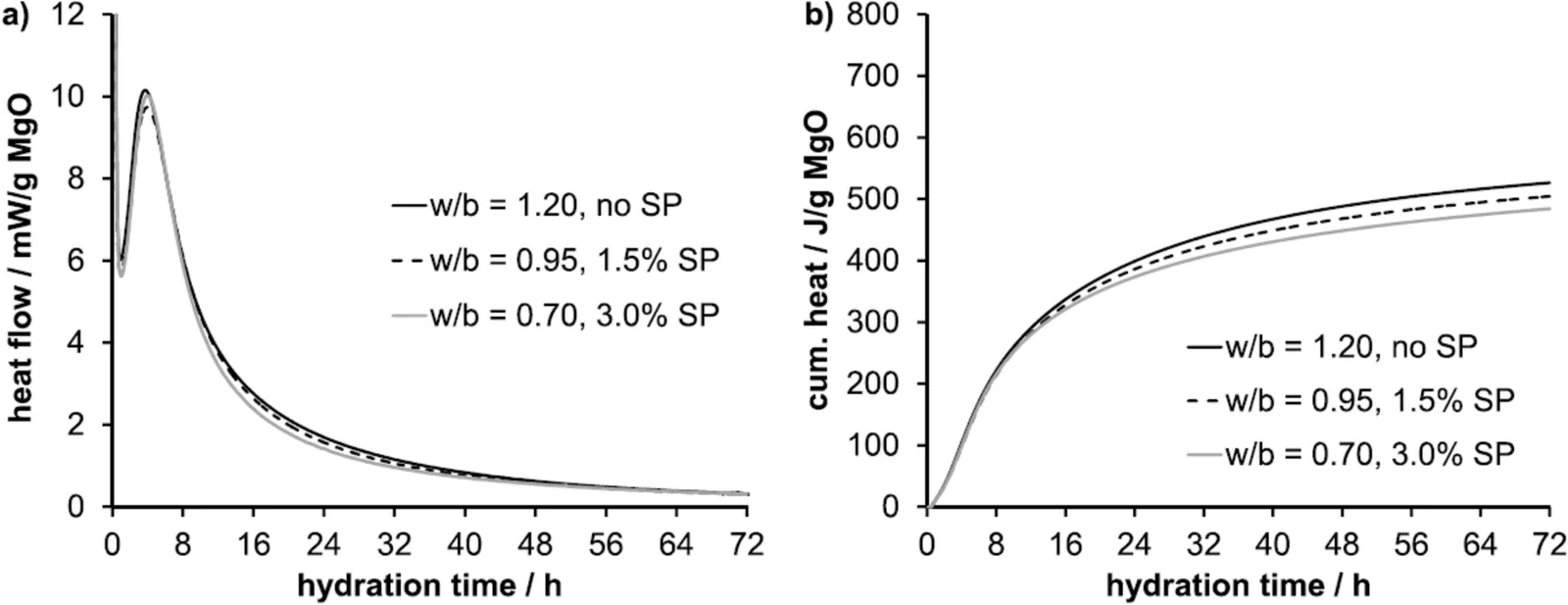
**a** Heat flow and **b** cumulative heat of MgO-hydromagnesite blend at different water/binder ratio and superplasticizers content. Reprinted from [17], with permission from Elsevier

In MgO/Nesquehonite systems, an acceleration of early hydration was observed for some mixes, which was eventually attributed to the effect of sodium ions present in the applied PCE [19]. The effect of PCEs on setting time and hydration was also investigated using MgO/hydromagnesite blends with medium-reactivity MgO [17]. High dosages of PCE superplasticizers (1.5–3 mass-% with 40% solid content) did not significantly delay hydration, as evidenced by isothermal calorimetry results (Fig. 5). Similar observations were made by [18].

In summary, it can be concluded, that in contrast to Portland cement-based systems, high PCE dosages (up to about 5 mass-% of the liquid PCE) do not delay hydration of magnesium carbonate cements to the same extent.

## Hardening accelerators

In magnesia-based binders, formation and reaction kinetics are among the key factors governing their effective use in construction. Unlike Ca^2+^, Mg^2+^ exhibits a strong coordinated hydration stage, creating a high energy barrier to its dehydration [87]. Additionally, the tight constraint in CO_3_^2−^ group [88] in anhydrous MgCO_3_ also contributes to energy-intensive MgCO_3_ formation. Therefore, admixtures are often used to facilitate and enhance the formation of different carbonate phases in the MgO–CO_2_–H_2_O and different silicates in the MgO–SiO_2_–H_2_O systems. At the time this paper is being drafted, the open literature does not have many reports on investigation of accelerators in magnesia-based cement at mortar or concrete levels but rather on fundamental research. However, there are several approaches that can potentially be applied as accelerators for magnesia-based cements in practical applications.

### Accelerators for magnesium silicate cement

Upon mixing magnesium silicate cements with water, the reactive MgO first hydrates to brucite, leading to a slightly alkaline pore solution [10]. Brucite subsequently reacts with silica to M-S-H, but at the given pH-value the solubility of brucite [14], and of silica, are low. Consequently, acceleration in magnesium silicate cements is mainly realized by promoting the formation of M-S-H by enhancing the dissolution of brucite and silica. So far, four categories of accelerators, sodium carbonates, alkali phosphates, sodium silicate and seeding, are identified.

#### Sodium carbonates

The addition of sodium carbonates such as Na_2_CO_3_ and NaHCO_3_ to a MgO-silica and/or brucite-silica system can accelerate the formation of M-S-H by enhancing the dissolution of brucite [89–91]. By forming magnesium carbonate complexes, sodium carbonate destabilizes brucite, increasing its dissolution rate [89].

**Fig. 6:**
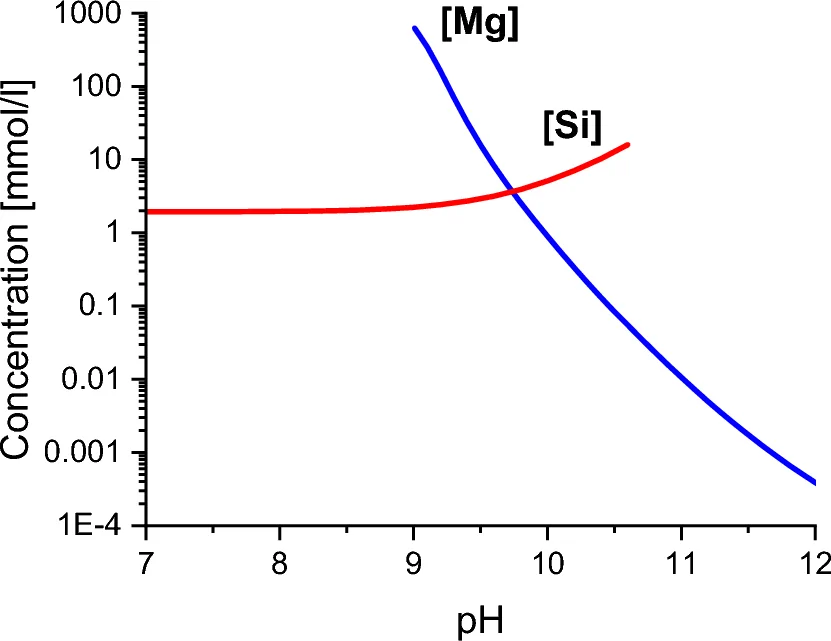
The solubility of amorphous silica and brucite in relation to pH in pure water. The calculation was done using the thermodynamic software GEMS [93, 94] coupled with PSI/Nagra [95] and Cemdata18 databases [96]. In the modelling, all solids, except amorphous silica and brucite are prohibited from forming

For acceleration in a brucite-silica system, a pH of ~ 10.2 (achieved by the addition of NaHCO_3_) is ideal [90]. This is due to the crossing of the opposing solubility of the two phases approximately at this pH range [92], as also predicted via thermodynamic calculation (Fig. 6).

Moreover, the addition of NaHCO_3_ results in the potential existence of not well crystallized hydromagnesite as an intermediate product, which increases the Mg^2+^ in solution and supports the silica dissolution, leading to increased amounts of M-S-H [91].

#### Alkali phosphates

Various alkali phosphates including NaHMP, NaOP, NaTMP and DKP have shown acceleration effects in magnesium silicate cements [10, 31, 33, 35]. The addition of NaHMP suppresses the formation of Mg(OH)_2_ [10, 97], resulting in increased Mg^2+^ concentration and pH. The latter enhances silica dissolution, and the dissolved silica reacts with Mg^2+^ and water to form M-S-H. The Mg(OH)_2_ inhibition is controlled by the Mg-phosphate speciation. With a strong complexation of Mg-phosphate, it was proposed that insoluble [6MgOH^+^·(PO_3_)_6_^6−^] precipitates on the MgO surface and blocks the nucleation site for Mg(OH)_2_. The concentration of NaHMP is critical to this process. While NaHMP concentrations below 1 mass-% do not fully inhibit brucite formation, concentrations between 2 and 5 mass-% effectively retard the dissolution of MgO [10].

**Fig. 7:**
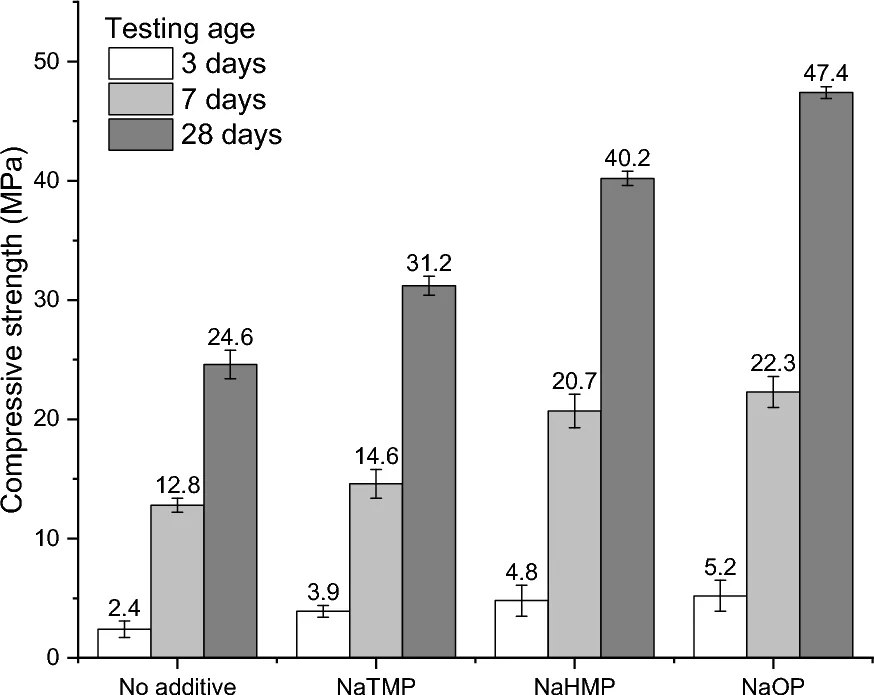
Compressive strength of magnesium silicate cement-based mortars with w/c ratio 0.40 containing 1.0 mass-% sodium phosphate additive (except for the reference). Based on data from [31]

In addition to NaHMP, NaOP and NaTMP enhance the formation of M-S-H without negatively effecting the structure of the phase, with NaOP being the most effective accelerator [31, 33]. NaHMP and NaTMP undergo dissolution resulting in mainly pyrophosphate and orthophosphate species of different proportions. A stronger acceleration was observed with higher amounts of orthophosphate species. This originates from the favoured interaction between this species and the MgO particles compared to pyrophosphate [33] as well as the higher Mg^2+^ concentration in combination with the higher silica reactivity [31]. The effect of the different sodium phosphates on the compressive strength development of a magnesium silicate cement is shown in Fig. 7.

DKP also enhances the degree of reaction of magnesium silicate cements and promotes the formation of M-S-H [35]. It was found that the compressive strength of M-S-H mixes with 3.0–5.5% DKP was superior to a reference M-S-H with 2% NaHMP. Performance at early age and at later ages showed opposite trends depending on the DKP dosage. Whereas the highest 3d strength was found for the lowest dosage (3%), the opposite was observed at 28d. The reaction of DKP with MgO led to the formation of K-struvite (MgKPO_4_ · 6H_2_O), which might have contributed to later age compressive strength by filling pore space. Additionally, it might serve as an intermediate for later magnesium supply for the M-S-H formation.

#### Sodium silicate

Sodium metasilicate was identified as effective accelerator by suppressing brucite formation [98]. Its addition leads to an increase in the dissolved Mg concentration. This, in combination with the elevated amounts of silicate species caused by the increased pH, promotes faster M-S-H formation during the first 24 h, bypassing the formation of Mg(OH)_2_. However, a side effect of this method is a change in microstructure from yolk-shell to flower-like and stacked sheet-like M-S-H structure. In [98], it was speculated that the flower-like morphology might increase the nucleation rate and formation of hydrates; further investigation on the nucleation and growth of M-S-H in this system is needed to confirm this observation.

#### Seeding

Seeding using hydromagnesite particles (BET: 43.5 m^2^/g) in a MgO-microsilica system has been shown to improve both the rate and degree of hydration [64]. The effect of the hydromagnesite was attributed to the provision of additional nucleation sites for M-S-H, enhancing the microstructural and strength development [64]. In addition to the seeding effect hydromagnesite can also accelerate the reaction of MgO through the intermediate formation of HCB [99] or brucite destabilization [89].

### Accelerators for magnesium carbonate cement

For magnesium carbonate cements, it seems that the availability of Mg^2+^ to readily react with carbonate ions is one of the key factors to form Mg-carbonates. Admixtures that promote this reaction have shown to be promising in literature. According to [100], a suitable admixture to facilitate the formation of Mg-carbonates will need to fulfil three key factors based on calculation at atomistic scale. First, the admixture is able to form solvent-shared ion pairs or contact ion pairs which are less stable than the contact ion pair Mg^2+^⋯CO_3_^2−^. Second, it can stabilize the undercoordinated state of Mg(H_2_O)_5_^2+^. Third, this additive facilitates the stability of Mg(*Additive*)(H_2_O)_3_^2^_3_^2–n^ and Mg(*Additive*)(H_2_O)_4_^2–n^ complexes where *n* is the valency of *Additive*. This offers a basic framework for scanning and searching suitable molecule structures to work as accelerators in MC cement. Suitable admixtures are expected to promote Mg^2+^ dehydration without inhibiting the formation of the Mg^2+^⋯CO_3_^2−^ contact ion pair which then potentially offer the enhanced formation of Mg-carbonates.

A common approach is to utilize organic admixtures such as mono-ethylene glycol [101] or acetate [102] to facilitate the formation of carbonate or hydrate phases. Mono-ethylene glycol is known to significantly reduce the water activity, enabling the synthesis of anhydrous MgCO_3_ at low temperature and pressure. Acetate increases the hydration of MgO, leading to the better availability of modified brucite for carbonation [103]. An increased conversion of MgO into brucite was also achieved with HCl [104]. Further, the use of enzymes such as carbonic anhydrase has been proven to be effective towards the formation of Mg-carbonates via the enhancement of the dissolution of CO_2_ in aqueous media [105–107] although their potential to upscale in construction materials remains an open question.

#### Magnesium acetate

**Fig. 8:**
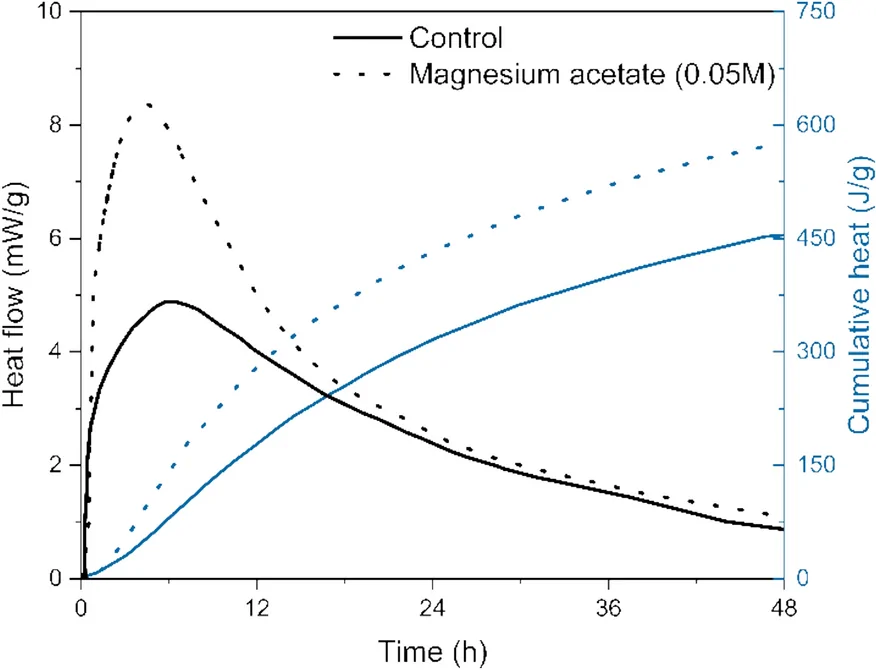
Effect of magnesium acetate as HA on initial and final setting time of MgO paste (adapted from [108])

Magnesium acetate has been shown to expedite the setting of MgO pastes [108]. With the addition of 0.05 M magnesium acetate, initial and final setting times of MgO paste were shortened by 16% and 20%, respectively (Fig. 8). It functioned as a hydration agent (HA), accelerating MgO dissolution by lowering the overall pH of the paste. Moreover, by providing more magnesium ions, magnesium acetate facilitated the formation of brucite in bulk solution for subsequent carbonation reactions.

The accelerating effect of magnesium acetate (0.05–0.1 M) was also evidenced by higher exothermic peaks and cumulative heat release in isothermal calorimetry reported in previous studies [36, 102, 109]. The exothermal peak in the heat evolution curve shifted from approximately 6 h to 4 h with the addition of 0.05 M magnesium acetate, accompanied by higher total heat release, see Fig. 8 [108].

 [103] observed higher exothermic peaks with increasing magnesium acetate concentration up to 0.5 M in MgO paste; however, at this concentration, initial cracks emerged in pastes (water/solid ratio of 1) due to complete hydration. The cumulative heat after 2 days of hydration at 0.05 M concentration was comparable to that at 0.1 M (around 940 J/g MgO), while that at 0.5 M concentration decreased to 905 J/g MgO, still higher than for the control sample (803 J/g MgO). The 0.5 M Mg-acetate sample also reached full reaction degree within 24 h of hydration which is in line with the cumulative heat from isothermal calorimetry. Therefore, for high-purity MgO, the estimation of reaction degree can be based on the cumulative heat. Furthermore, it is critical to determine the optimum concentration of magnesium acetate to achieve the desired acceleration effect on the setting time and rheological properties of MC cement.

#### Microbial acceleration

**Fig. 9:**
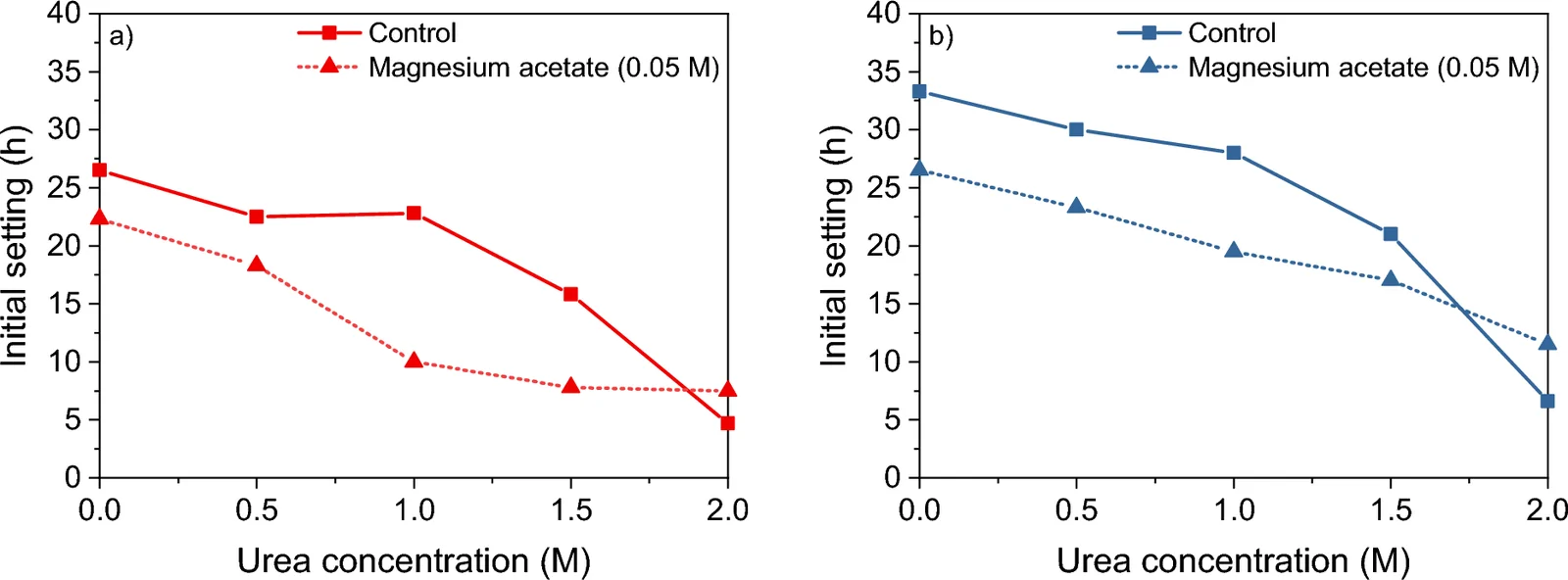
Effect of urea concentration and HA on **a** initial and **b** final setting time of MgO paste incorporating urease-producing bacteria (based on [108])

The use of microbes has also demonstrated potential to accelerate carbonation in MC cements, reducing setting times [108, 110]. In the presence of urease-producing bacteria (optical density OD600:1.7; urease activity: 15 U/ml), increasing the urea concentration from 0.5 to 2 M led to a decrease in the initial and final setting time of MgO paste by 79% and 78%, respectively (Fig. 9). This was attributed to the urea hydrolysis catalysed by urease from bacteria, which generated additional carbonate ions and promoted carbonation reactions.

Furthermore, the synergistic effect of urea concentration and magnesium acetate as HA on MgO setting was also investigated [108], as shown in Fig. 9. The use of HA reduced the initial and final setting time of MgO paste containing urease-producing bacteria at urea concentration ranging from 0.5 to 1.5 M, except for 2 M. At 2 M concentration, the accelerating effect resulting from urea hydrolysis took precedence, whereas the lower pH from magnesium acetate potentially inhibited bacterial activity. Therefore, microbial approaches could have an accelerating effect on MC through self-carbonation, however, the performance depends on the dosage of the bacteria and nutrients used, as well as bacteria activity.

## Retarders

The control of setting and workability time by retarding admixtures is crucial for almost all applications of mortars and concretes, especially when the material is not placed immediately after mixing. However, there is comparatively little research on retarders and their mechanisms in magnesia-based cements [1]. Most of the research so far has focused on magnesium phosphate cements, which are outside the scope of this article. Nevertheless, the reactive MgO used in magnesium silicate and magnesium carbonate cements may react rather fast, which may result in early stiffening and rapid setting. For instance, recent laboratory trials on concretes with a binder based on reactive MgO and hydromagnesite showed a rapid loss of slump flow (measured with the flow table test according to EN 12350-5) during the first 10 min after mixing [25, 26]. Isothermal calorimetry confirmed the very fast reaction of such binders [16–19, 25], making a control of the setting time crucial for application. On the contrary, binders based on magnesium silicate often react rather slowly [1], and retardation might be less an issue, probably depending on the reactivity of the MgO used.

### Retarders for magnesium silicate cements

Theoretically, the retardation of M-S-H formation can be approached by the following strategies: a) hindering the dissolution of MgO or SiO_2_; b) changing the pH to values outside the ideal pH range (9–11.5) for M-S-H formation [111]. The retardation of magnesium silicate cement, however, is generally not favourable for many applications, due to its already long hardening process. Therefore, only a few studies about the use of high dosages of phosphates and immobilization of heavy metal cations have reported retardation effects.

#### Alkali phosphates

Generally, NaHMP can accelerate the formation of M-S-H by suppressing the precipitation of Mg(OH)_2_. However, when phosphate concentrations exceed 2 mass-%, a retarding effect on the dissolution of MgO and the formation of M-S-H has been observed. This can be attributed to two factors, (i) a large number of negative phosphate ions or Mg phosphate complexes [6MgOH^+^·(PO_3_)_6_^6−^] are adsorbed on the positively charged MgO surface, which hinders the further dissolution of MgO [10], and (ii) the strong Mg-phosphate complexes lower the concentration of free Mg ions in the solution, thereby hindering the formation of M-S-H [111, 112].

#### Heavy metal cations

**Fig. 10:**
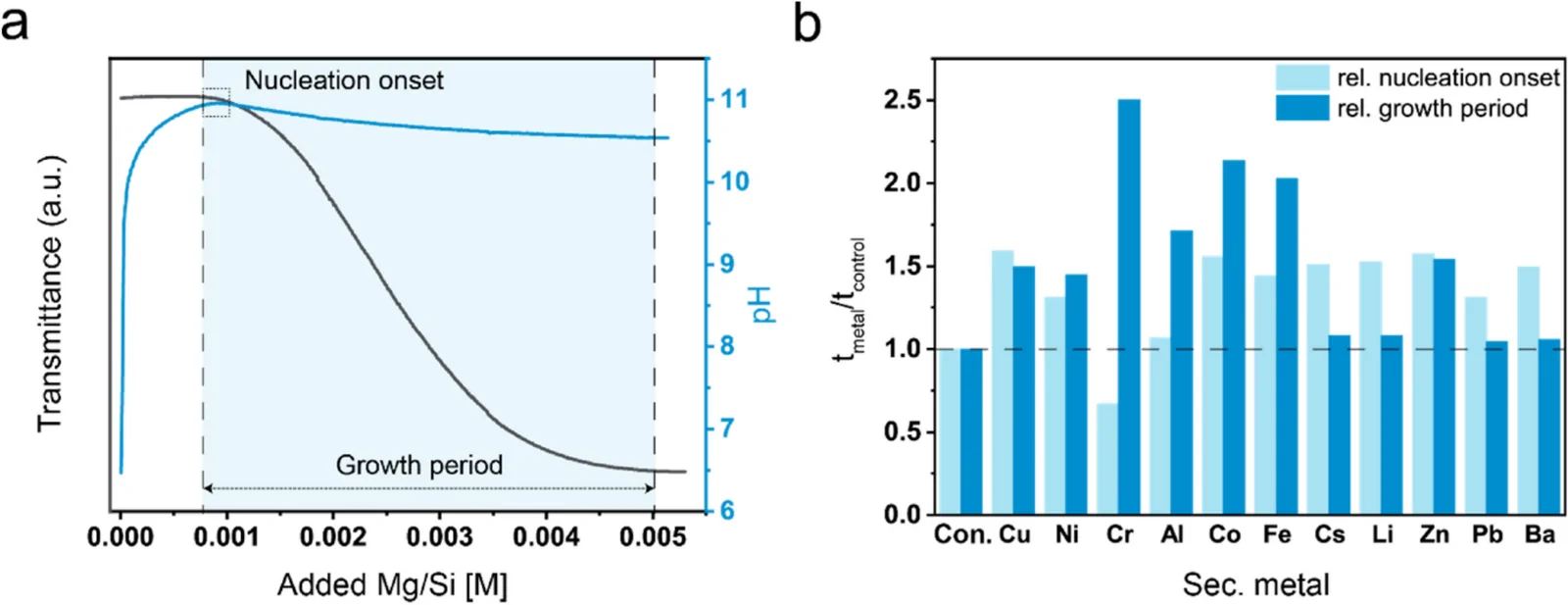
**a** Example for pH (blue) and transmission curves (black) during the titration experiment; **b** M-S-H nucleation onset time and growth period normalized to the control group without any addition of heavy metal cations. Figures taken from [113] under the terms and conditions of the Creative Commons Attribution (CC BY) license (http://creativecommons.org/licenses/by/4.0/). (colour figure online)

In investigations on the immobilization of heavy metal cations in M-S-H cement through titration experiments [113], the authors found that the addition of Fe^3+^, Co^2+^ and Cr^3+^ as chlorides causes a retardation of M-S-H growth (Fig. 10) and leads to higher heavy metal uptake in the final M-S-H phase. Although the primary purpose of the studies was to investigate the immobilization, nevertheless important insights on retardation can be drawn from them. An immobilization mechanism was suggested, where heavy metal cations form complexes with hydroxyl ions, producing hydroxide complexes that are incorporated in the M-S-H matrix. Therefore, the formation of heavy metal hydroxides consumes hydroxyl ions in the solution, prolonging the formation of M-S-H. A similar retardation effect on M-S-H was reported in the presence of Sr^2+^ cations [59], which is explained by the possible competition of hydroxyl ions (OH^−^) for the formation of M-S-H and the formation of Sr(OH)_2_, leading to less abundant hydroxyl ions for the formation of M-S-H. Therefore, the retardation caused by those heavy metal cations is linked to the competition of hydroxyl ions (OH^−^) or buffering of the pH to slow down the formation of M-S-H.

### Retarders for magnesium carbonate cements

To date, research has not focused on retarders for magnesium carbonate cements, but rather for pure magnesium oxide (MgO). The primary purpose of retarders in magnesium carbonate cements is to delay the hydration of MgO, and subsequently, the formation of brucite (Mg(OH)_2_). There are two main approaches to slow down the formation of brucite (i) reducing the dissolution of MgO and/or (ii) hindering the precipitation of brucite [114]. Currently, two main types of retarders have been investigated: alkali phosphates, particularly, NaHMP and organic retarders, namely citric acid and sodium gluconate.

#### Alkali phosphates

NaHMP is widely used in several industries as deflocculant to disperse particles in suspension [10]. It is also known as a retarder for brucite precipitation during the MgO hydration process [97]. According to X-ray diffraction results after 7 days, a limited amount of brucite was formed in mixes containing NaHMP compared to the control mix without NaHMP [10]. Moreover, a clear retardation of MgO dissolution was observed in samples with more than 2 mass-% of NaHMP.

**Fig. 11:**
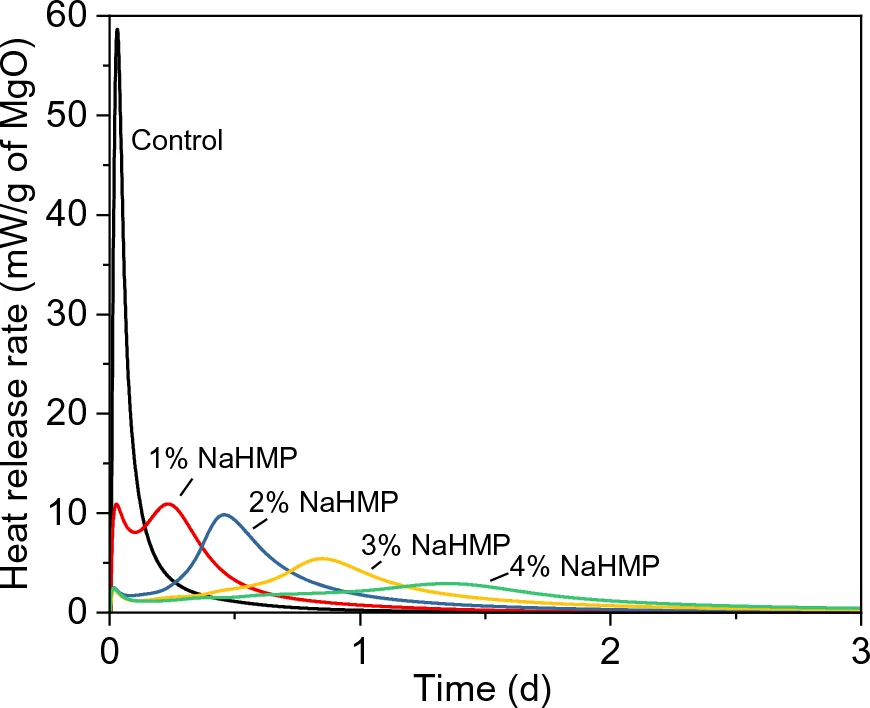
Isothermal calorimetry of magnesia-based paste with varying amounts of NaHMP in mass-%, adapted from [68]

The retarding effect of NaHMP was also demonstrated by [68], where both the initial and final setting time of a magnesia-based paste were significantly longer when NaHMP was added. The early hydration behaviour of MgO has also been studied using isothermal calorimetry (Fig. 11). In [68], the addition of NaHMP resulted in a double-peak curve, with an initial sharp peak which is believed to correspond to the initial dissolution of MgO, followed by a broader main peak. As the dosage of NaHMP increased, the first peak was strongly reduced. The second peak was shifted to later times, and its shape became progressively broader with a lower maximum.

The effect of NaHMP (1 mass-%) on the hydration of MgO-binder was studied with a combination of multiple methods [104]. Though with NaHMP a retarding effect on early hydration was also observed by isothermal calorimetry, the degree of hydration (brucite formation) from the age of 3 days onwards was similar for the pure MgO-binder and the mix with NaHMP. Furthermore, NaHMP had a positive effect on the compressive strength, especially under accelerated carbonation curing [104]. It should be noted, however, that the water-cement ratio was lowered in the NaHMP containing mix, thus the change in water content influenced both the hydration rate and the strength development.

Recently, also sodium orthophosphate was studied as possible retarder for magnesium carbonate cements [115]. It was found that phosphate ions sorbed on the surfaces of MgO particles which retarded dissolution of MgO and formation of brucite. Phosphate was incorporated in magnesium phosphate and brucite-like phases. While for pure MgO a significant retardation could be observed, the effect on MgO-nesquehonite blends was less pronounced.

#### Organic retarders

**Fig. 12:**
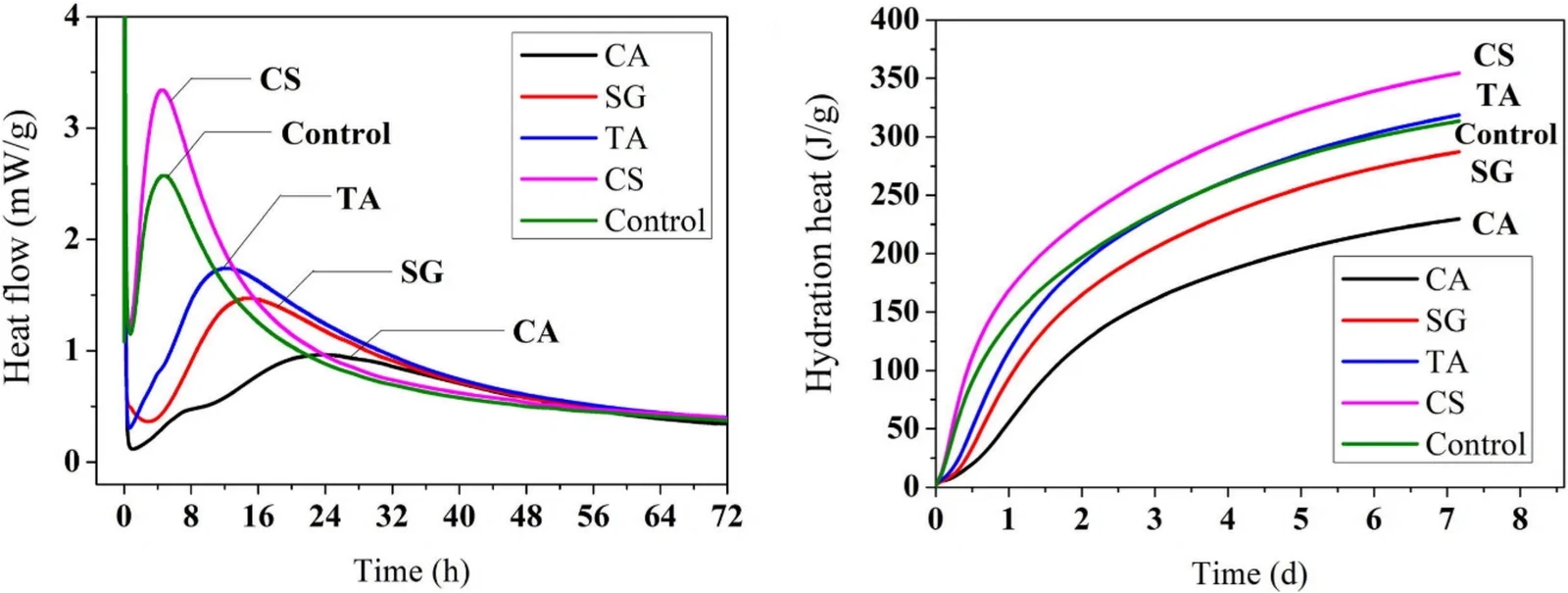
Effect of retarders on hydration heat release of MgO in different time (water/solid = 1, 0.1 mass-% of retarders). CA = citric acid, CS = calcium sulphate, SG = sodium gluconate, TA = tartaric acid. Reprinted from [117], with permission from Elsevier

**Fig. 13:**
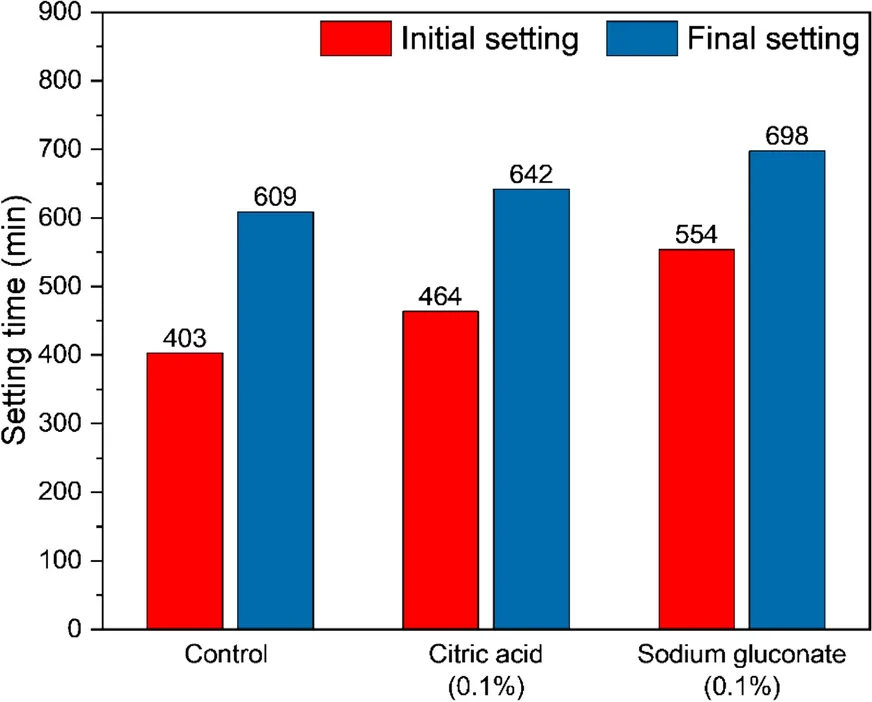
Effect of citric acid and sodium gluconate on initial and final setting time of MgO pastes (drawn from data presented in [117])

A retarded formation of Mg(OH)_2_ from MgO can be achieved by adding citric acid (C_6_H_8_O_7_). In water, citric acid dissociates to form negatively charged citrate ions. With their maximum three negative sites, citrate ions can be adsorbed on the positively charged surface of MgO, which is suggested to slow down the hydration of MgO [114, 116]. Moreover, because of its high affinity for metal cations such as Mg^2+^, citric acid can complex the dissolved Mg^2+^ ions, which can therefore inhibit the precipitation of brucite [114]. It has been shown by [117] that citric acid extends both the induction and the acceleration period (Fig. 12), and reduces the hydration degree at early stage. Addition of 0.1% of citric acid by weight of binder showed prolonged initial and final setting time by approximately 60 and 30 min (Fig. 13), respectively, while 0.3% was found sufficient to strongly slow down hydration of MgO on the long-term [114, 118]. It has also been shown that citric acid improves the long-term compressive strength, which makes citric acid a promising retarder for magnesia-based cements [117].

The use of sodium gluconate (C_6_H_11_O_7_Na) has also been reported to have a retarding effect on MgO hydration [117]. 0.1% of sodium gluconate by weight of binder was sufficient to extend the induction period of MgO hydration (Fig. 12), as well as the initial and final setting time by approximately 150 and 90 min (Fig. 13), respectively. According to [117], the prolongation of the induction period could be due to the surface adsorption and the hindering of nucleation and growth of brucite. Besides, the hydration degree at early age is also reduced [117]. However, a detrimental effect on the compressive strength after 28 days was observed [117]. Moreover, sodium gluconate is also known mainly as a retarder with some water-reducing properties for Portland cement, and the effect might be similar for magnesia-based binders [119].

## Other admixtures

Information on the use of admixtures other than plasticizers, accelerators and retarders in magnesia-based binders is scarce, which shows that there is a significant gap in the research in this area. The following few examples indicate that admixtures for PC-based concrete can also be effective in magnesia-based concretes, mortars or pastes.

### Air entrainers

In [120], an air entraining agent based on sodium lauryl sulphate was used for the production of pastes either with PC or with reactive MgO cement subjected to carbonation hardening. Using the foam index test, a higher stability of the entrained air in the MgO-paste was found. The entrained air increased the workability of the paste and had a positive effect on the long-term compressive strength, as it facilitated CO_2_ diffusion. In [28], the use of an air entraining agent in MgO-metakaolin-based concrete is described. However, details on the specific purpose or effectiveness of the agent were not provided.

### Shrinkage reducing admixtures

The effect of a superabsorbent polymer (SAP) as an internal curing agent to mitigate autogenous shrinkage in magnesium silicate pastes was studied by [121]. The SAP was described as a low-crosslinking sodium polyacrylate with median particle size of 373 µm and water absorption (teabag method) of 350 g/g. When 0.5% by weight of binder were added to magnesium silicate cement without an additional amount of water, the SAP was found to significantly reduce autogenous shrinkage.

Zhang et al. [60] studied the effectiveness of a shrinkage reducing admixture, which was described as “alkyl polyether organic substance”, on the autogenous and drying shrinkage of a magnesium silicate cement mortar. The admixture was more effective in reducing autogenous than drying shrinkage. A detrimental effect of hydration, compressive strength development and resulting porosity was observed.

## Conclusion and suggestions for future work

The production of concrete with magnesia-based binders remains challenging, even at laboratory scale. A primary challenge is ensuring good workability, and another is achieving adequate strength development. Though significant progress has been made through intensive research, there remain substantial gaps in understanding the role of admixtures in magnesia-based binders. In particular, there is little research available on magnesium carbonate cements, as many studies have been conducted only on model systems based on MgO.

### Plasticizers

Alkali phosphates are a very effective plasticizer for magnesia-based binders, especially for magnesium silicate cements with the additional benefits of retarding the setting and improving the hardening. Though many different alkali phosphates exist, only a few have been tested for their effectiveness. Among these, sodium hexametaphosphate (NaHMP) is the most prominent, but not necessarily the most effective one. There is also uncertainty as to whether genuine sodium hexametaphosphate was always used when declared, as high-polymeric polyphosphate mixtures are often sold under the commercial name sodium hexametaphosphate [43]. The effect of different types of alkali phosphate on workability should be addressed systematically in future studies. Experience from other industrial applications indicates that the degree of condensation (e.g. chain length) of alkali phosphates can be a decisive parameter for its dispersing effect [43].

Based on experiments with NaHMP, a recent study proposed a dispersion mechanism for alkali phosphate admixtures in magnesia-based binder systems. Accordingly, NaHMP undergoes hydrolysis and linear polyphosphate chains are adsorbed on the particle surfaces. Thereby, positive surface charges are neutralized and negative surface charges are increased, leading to enhanced electrostatic repulsion [47]. It would be valuable to validate these results also using other alkali phosphates, especially polyphosphates.

Limited studies have examined the effect of the alkali phosphate dosage on rheology, highlighting the need for additional research in this area, particularly considering the presence of a threshold dosage beyond which the dispersing effect is reduced again. Understanding the cause for this threshold as well as the parameters which affect it, could be crucial for practical applications in the industry. In this regard, different magnesia-based binder types as well as different qualities (e.g. lab grade vs. industrial grade) should be studied. Furthermore, there is still a gap in knowledge regarding the possible formation of magnesium phosphate phases and their effect on rheological properties.

Polycarboxylate ether (PCE)-based plasticizers have also been shown to be effective for magnesia-based binders. However, due to the high-water demand of these binders, higher PCE-dosages than for Portland cement are required to achieve a workable mix. Similar to alkali phosphates, there is an optimal dosage of PCE for improving rheology, and excessive amounts may lead to issues such as bleeding and segregation. Furthermore, the improvement of existing or the development of new plasticizers and dispersants, tailored to the use in magnesia-based binder systems, can help to enable the practical application of these binders.

### Accelerators

In magnesium silicate cements, alkali phosphates have proven to be a good hardening accelerator, as they can hinder brucite formation and facilitate the reaction of dissolved magnesia and silica. Similar to their use as plasticizer, open questions remain regarding the most effective type and/or dosage of the alkali phosphates. This should be also evaluated in combination with different MgO and silica types and reactivities. Ideally, a fundamental understanding of the interaction of the different parameters should be sought. Accelerating effects with magnesium silicate cement were also reported for sodium carbonate, sodium silicate, and hydromagnesite seeds.

For magnesium carbonate cements, it seems that the availability of Mg^2+^ to readily react with carbonate ions is one of the key factors to form Mg-carbonates. Admixtures that promote this reaction have shown to be promising in literature, e.g. organic admixtures such as magnesium acetate or mono-ethylene glycol. Other approaches for accelerators include the use of HCl or microbial accelerators.

Future studies should focus on admixtures which are expected to promote Mg^2+^ dehydration without inhibiting the formation of the Mg^2+^⋯CO_3_^2−^ contact ion pair, as these potentially offer the enhanced formation of Mg-carbonates. Anion additives that fulfil all mentioned criteria, such as sulphate, dihydrogen phosphate, oxalate, citrate, malate, and aminophenolate, may be worth testing at cement mortar and concrete level or implementing in additive development processes.

### Retarders

While retarders may not be necessary for magnesium silicate cements (unless the MgO is very reactive), they are crucial for magnesium carbonate cements, as rapid workability loss and fast setting are reported for these systems. In the very few studies on pure MgO reported so far, either phosphates or organic hydroxy-carboxylic acids (and their salts) such as citrate and gluconate show a potential to be suitable retarders. However, their mechanisms of action need a more detailed investigation, and they should not compromise strength development or volume stability. Studies on further potential retarders are needed as well, i.e. including chelating and complexing agents other than citrate, gluconate, and phosphate. HCB (hydrous-carbonate-containing brucite), the principal hydration product of magnesium carbonate cement [18, 20], contains (bi)carbonate ions which are supplied by reactive HMCs such as hydromagnesite and nesquehonite as part of the binder. So far, open literature does not report work on retarders for such systems, where an interaction with the carbonate/bicarbonate system will play an important role.

### Other admixtures

Only very limited data exists here for magnesia-based binder systems. A fundamental evaluation towards the effectiveness of several kinds of admixtures besides plasticizers, accelerators and retarders is necessary. For practical applications in infrastructure, the focus should be placed on air entraining agents as a means to improve the frost and salt-frost resistance of concrete. Though it was shown by [28, 120] that air-entrainment is generally possible with magnesia-based binders, no systematic research has been conducted with regard to effective dosages, required mixing times, and air void stability for different types of air-entraining agents and binders. Furthermore, the effectiveness of air-void systems to obtain frost and salt-frost resistant concrete with magnesia-based binders still remains to be studied.

It has been proposed by [28], that a self-compacting consistency is favourable for concrete with magnesia-based binders in order to cope with their high viscosity. The application of stabilizers could therefore be beneficial to ensure sedimentation resistance. The effect of stabilizers on rheological properties and sedimentation as well as on strength development and durability has not been studied as yet.

As previous studies have indicated that drying shrinkage cracking can occur in magnesia-based binder systems [60, 65], shrinkage reducing admixtures could be applied for mitigation. A systematic study on the effectivity of existing (or new) types of shrinkage reducers for the different types of magnesia-based binders is still missing. This should include the effect on autogenous and drying shrinkage, but also possible negative effects on hydration, strength development and porosity.

## Footnotes

1 Acronyms for alkali phosphates found in literature are not uniform. For clear identification, the acronyms used in the present paper always include the type of alkali—Na for sodium and K for potassium—in the compound.

## References

[1] Bernard E, Nguyen H, Kawashima S, Lothenbach B, Manzano H, Provis J, Scott A, Unluer C, Winnefeld F, Kinnunen P (2023) MgO-based cements—current status and opportunities. RILEM Tech Lett 8:65–78. 10.21809/rilemtechlett.2023.177

[2] Lothenbach B, Bernard E, German A, Winnefeld F (2023) MgO-based binders. ce/papers 6:342–356. 10.1002/cepa.2774

[3] Lothenbach B, Bernard E, Zhou Z, German A, Montserrat-Torres P, Winnefeld F (2026) Critical review of the properties of MgO—magnesium carbonate cements. Cem Concr Res 200:108092. 10.1016/j.cemconres.2025.108092

[4] Walling SA, Provis JL (2016) Magnesia-based cements: a journey of 150 years, and cements for the future? Chem Rev 116:4170–4204. 10.1021/acs.chemrev.5b00463 27002788

[5] Mundra S, Hallet V, Bernard E, Ilango NK, Nguyen H, Furcas FE, Ranjbar N, Meng D, Gevauden JP, Mueller M, Aiken T, Lothenbach B (2026) Durability of MgO-based binders: a critical review from RILEM TC 311-MBC. Mater Struct 59:202. 10.1617/s11527-026-03076-7 PMC13680333 42903152

[6] Bernard E, Yio M, Rentsch D, Chen H, Myers RJ (2024) Insights on the effects of carbonates and phosphates on the hydration of magnesia (alumino-)silicate cements. Appl Geochem 167:106001. 10.1016/j.apgeochem.2024.106001

[7] Scott A, Oze C, Shah V, Yang N, Shanks B, Cheeseman C, Marshall A, Watson M (2021) Transformation of abundant magnesium silicate minerals for enhanced CO_2_ sequestration. Commun Earth Environ 2:25. 10.1038/s43247-021-00099-6

[8] Scott AN, Shah V, Oze C, Shanks B, Cheeseman C (2021) Use of olivine for the production of MgO-SiO_2_ binders. Front Built Environ 7:640243. 10.3389/fbuil.2021.640243

[9] Naber C, Majzlan J, Moosdorf N, Neubauer J, Wagner D, Bellmann F (2026) Magnesium silicate binder shows potential as a carbon-neutral route for cement manufacture. Commun Sustain 1:79. 10.1038/s44458-026-00085-z

[10] Jia Y, Wang B, Wu Z, Han J, Zhang T, Vandeperre LJ, Cheeseman CR (2016) Role of sodium hexametaphosphate in MgO/SiO_2_ cement pastes. Cem Concr Res 89:63–71. 10.1016/j.cemconres.2016.08.003

[11] Wei J, Chen Y, Li Y (2006) The reaction mechanism between MgO and microsilica at room temperature. J Wuhan Univ Technol Mater Sci Ed 21:88. 10.1007/BF02840848

[12] Zhang T, Vandeperre LJ, Cheeseman CR (2012) Magnesium-silicate-hydrate cements for encapsulating problematic aluminium containing wastes. J Sustain Cem Based Mater 1:34. 10.1080/21650373.2012.727322

[13] Walling SA, Kinoshita H, Bernal SA, Collier NC, Provis JL (2015) Structure and properties of binder gels formed in the system Mg(OH)_2_–SiO_2_–H_2_O for immobilisation of Magnox sludge. Dalton Trans 44:8126. 10.1039/C5DT00877H 25833071

[14] Gevaudan JP, Ruiz-Agudo C, Manzano H, Nguyen H, Lothenbach B, Kinnunen P, Bernard E (2026) Dissolution, diffusion, and precipitation of Mg2+ in MgO-based cements, a review by the RILEM TC-311 MBC. Mater Struct 59:161. 10.1617/s11527-026-03048-x PMC13668919 42889278

[15] Dhakal M, Scott AN, Shah V, Dhakal RP, Clucas D (2021) Development of a MgO-metakaolin binder system. Constr Build Mater 284:122736. 10.1016/j.conbuildmat.2021.122736

[16] Kuenzel C, Zhang F, Ferrándiz-Mas V, Cheeseman CR, Gartner EM (2018) The mechanism of hydration of MgO-hydromagnesite blends. Cem Concr Res 103:123–129. 10.1016/j.cemconres.2017.10.003

[17] Winnefeld F, Epifania E, Montagnaro F, Gartner E (2019) Further studies of the hydration of MgO-hydromagnesite blends. Cem Concr Res 126:105912. 10.1016/j.cemconres.2019.105912

[18] German A, Winnefeld F, Lura P, Rentsch D, Lothenbach B (2023) Hydrous carbonate-containing brucite (HCB) in MgO/hydromagnesite blends. Cem Concr Res 173:107304. 10.1016/j.cemconres.2023.107304

[19] Montserrat-Torres P, Winnefeld F, Lothenbach B (2025) Impact of nesquehonite on hydration and strength of MgO-based cements. Cem Concr Res 189:107772. 10.1016/j.cemconres.2024.107772

[20] Jansen D, German A, Ectors D, Winnefeld F (2024) Stacking faults of the hydrous carbonate-containing brucite (HCB) phase in hydrated magnesium carbonate cements. Cem Concr Res 175:107371. 10.1016/j.cemconres.2023.107371

[21] Zhang T, Cheeseman CR, Vandeperre LJ (2011) Development of low pH cement systems forming magnesium silicate hydrate (M-S-H). Cem Concr Res 41:439. 10.1016/j.cemconres.2011.01.016

[22] Tran HM, Scott A (2017) Strength and workability of magnesium silicate hydrate binder systems. Constr Build Mater 131:526–535. 10.1016/j.conbuildmat.2016.11.109

[23] Zhang T, Vandeperre LJ, Cheeseman CR (2014) Formation of magnesium silicate hydrate (M-S-H) cement pastes using sodium hexametaphosphate. Cem Concr Res 65:8–14. 10.1016/j.cemconres.2014.07.001

[24] Wei J, Yu Q, Zhang W, Zhang H (2011) Reaction products of MgO and microsilica cementitious materials at different temperatures. J Wuhan Univ Technol Mater Sci Ed 26:745. 10.1007/s11595-011-0304-3

[25] German A (2024) Potential use of a low-carbon magnesia (MgO) binder for construction purposes. PhD Thesis, ETH Zürich, Switzerland

[26] Lura P, German A, Toropovs N, Sirtoli D, Winnefeld F (2026) Mechanical properties and volume changes of structural concrete with MgO/hydromagnesite binder. Mater Struct. 10.1617/s11527-026-03087-4

[27] Dhakal M, Scott AN, Dhakal RP, Clucas D (2022) Structural and durability properties for magnesia alumina silicate concrete. Constr Build Mater 340:127725. 10.1016/j.conbuildmat.2022.127725

[28] Dhakal M, Scott AN, Dhakal RP, Sullivan TJ, Clucas D (2022) Performance of MgO-Mk based MSH concrete column reinforced with steel and BFRP bars under cyclic loads. Structures 46:1764–1780. 10.1016/j.istruc.2022.11.025

[29] Tran H, Dhakal R, Scott A (2020) Mechanical and durability properties of magnesium silicate hydrate binder concrete. Mag Concr Res 72:693–702. 10.1680/jmacr.18.00217

[30] Zhang T (2012) Development of novel low pH Magnesium Silicate Hydrate (M-S-H) cement systems for encapsulation of problematic nuclear wastes*.* Dissertation, Imperial College London

[31] Kumar S, Lei J, Yang E-H, Unluer C (2022) Influence of different additives on the rheology and microstructural development of MgO–SiO_2_ mixes. Compos B Eng 235:109784. 10.1016/j.compositesb.2022.109784

[32] Sonat C, Unluer C (2017) Investigation of the performance and thermal decomposition of MgO and MgO-SiO_2_ formulations. Thermochim Acta 655:251–261. 10.1016/j.tca.2017.07.009

[33] Tonelli M, Martini F, Milanesi A, Calucci L, Geppi M, Borsacchi S, Ridi F (2019) Effect of phosphate additives on the hydration process of magnesium silicate cements. J Therm Anal Calorim 138:3311–3321. 10.1007/s10973-019-08847-9

[34] Zhang D, Wu H, Li VC, Ellis BR (2020) Autogenous healing of Engineered Cementitious Composites (ECC) based on MgO-fly ash binary system activated by carbonation curing. Constr Build Mater 238:117672. 10.1016/j.conbuildmat.2019.117672

[35] Li Z, Lin L, Yu J, Tang H, Qin J, Qian J (2022) Performance of magnesium silicate hydrate cement modified with dipotassium hydrogen phosphate. Constr Build Mater 323:126389. 10.1016/j.conbuildmat.2022.126389

[36] Dung NT, Unluer C (2022) Potential additives for magnesia-based concrete with enhanced performance and propensity for CO_2_ sequestration. J CO2 Util 56:101834. 10.1016/j.jcou.2021.101834

[37] Ruan S, Qiu J, Yang E-H, Unluer C (2018) Fiber-reinforced reactive magnesia-based tensile strain-hardening composites. Cem Concr Compos 89:52–61. 10.1016/j.cemconcomp.2018.03.002

[38] Tang Y, Qiu J (2024) CO_2_-sequestering ability of lightweight concrete based on reactive magnesia cement and high-dosage biochar aggregate. J Clean Prod 451:141922. 10.1016/j.jclepro.2024.141922

[39] Wu B, Chung YW, Tang Y, Qiu J (2024) Effect of internal moisture content (IMC) on the CO_2_ sequestration efficiency of hollow natural fiber (HNF)-reinforced reactive magnesia cement (RMC) composites. Constr Build Mater 428:136339. 10.1016/j.conbuildmat.2024.136339

[40] Wu B, Qiu J (2022) Incorporating hollow natural fiber (HNF) to enhance CO_2_ sequestration and mechanical properties of reactive magnesia cement (RMC)-based composites: Feasibility study. J CO2 Util 57:101874. 10.1016/j.jcou.2021.101874

[41] Wu B, Wang P, Qiu J (2024) Autogenous healing of the interface between hollow natural fiber (HNF) and reactive magnesia cement (RMC) matrix. Constr Build Mater 422:135843. 10.1016/j.conbuildmat.2024.135843

[42] Wu B, Wang P, Su X, Li W, Leung CKY, Qiu J (2024) The effect of depth-dependent carbonation on the interfacial strength between GFRP reinforcement bar and reactive magnesia cementitious composites. Cem Concr Compos 150:105533. 10.1016/j.cemconcomp.2024.105533

[43] Schrödter K, Bettermann G, Staffel T, Klein T, Hofmann T (2005) Phosphoric acid and phosphates*.* Ullmann's Encyclopedia of Industrial Chemistry

[44] Thilo E, Wieker W (1957) Zur Chemie der kondensierten Phosphate und Arsenate. Die Anionenhydrolyse kondensierter Phosphate in verdünnter wäßriger Lösung. Z Anorg Allg Chem 291:164–185. 10.1002/zaac.19572910120

[45] Gill JB, Riaz SA (1969) Kinetics of degradation of long chain polyphosphates. J Chem Soc A Inorg Phys Theor 4:183–187. 10.1039/J19690000183

[46] Watanabe M, Matsuura M, Yamada T (1981) The mechanism of the hydrolysis of polyphosphates. V. The effect of cations on the hydrolysis of pyro- and triphosphates. Bull Chem Soc Jpn 54:738–741. 10.1246/bcsj.54.738

[47] Kuhn R, Lura P, Rentsch D, Habert G, Bernard E (2026) Dispersant mechanisms in clays stabilized with MgO-based cement. Cem Concr Res 199:108040. 10.1016/j.cemconres.2025.108040

[48] Odegard C, Feldborg H, Myhre B (2001) Magnesia-silica-hydrate bonded MgO castables. In: Unified International Technical Conference of Refractories (UNITECR`01). Cancún, Mexico

[49] Canterford JH (1985) Magnesia - an important industrial mineral: a review of processing options and uses. Miner Process Extr Metall Rev 2:57

[50] Peng Y, Unluer C (2024) Evaluation of rheo-viscoelasticity of magnesium-silicate-hydrate mixes incorporating different superplasticizers. Dev Built Environ 20:100544. 10.1016/j.dibe.2024.100544

[51] Panda B, Sonat C, Yang E-H, Tan MJ, Unluer C (2021) Use of magnesium-silicate-hydrate (M-S-H) cement mixes in 3D printing applications. Cem Concr Compos 117:103901. 10.1016/j.cemconcomp.2020.103901

[52] Lin H, Pan B, Li Y, Luo X, Da D (2024) The effect of Mg(OH)_2_ on hydrated magnesium silicate cement under different curing conditions. Constr Build Mater 445:137799. 10.1016/j.conbuildmat.2024.137799

[53] Peng Y, Unluer C (2024) Performance and microstructural development of 3D printable MgO-SiO_2_ mixes containing magnesium silicate monohydrate. Ceram Int 50:33984–33994. 10.1016/j.ceramint.2024.06.218

[54] Sonat C, Teo WW, Unluer C (2018) Performance and microstructure of MgO-SiO_2_ concrete under different environments. Constr Build Mater 184:549–564. 10.1016/j.conbuildmat.2018.07.032

[55] Sonat C, Unluer C (2019) Development of magnesium-silicate-hydrate (M-S-H) cement with rice husk ash. J Clean Prod 211:787–803. 10.1016/j.jclepro.2018.11.246

[56] Zhang T, Dieckmann E, Song S, Xie J, Yu Z, Cheeseman C (2018) Properties of magnesium silicate hydrate (M-S-H) cement mortars containing chicken feather fibres. Constr Build Mater 180:692–697. 10.1016/j.conbuildmat.2018.05.292

[57] Zhang T, Du Y, Sun Y, He Z, Wu Z (2016) Development of magnesium-silicate-hydrate cement by pulverized fuel ash. Key Eng Mater 709:61. 10.4028/www.scientific.net/KEM.709.61

[58] Zhang T, Li T, Zhou Z, Li M, Jia Y, Cheeseman C (2020) A novel magnesium hydroxide sulfate hydrate whisker-reinforced magnesium silicate hydrate composites. Compos B Eng 198:108203. 10.1016/j.compositesb.2020.108203

[59] Zhang T, Zou J, Li Y, Jia Y, Cheeseman CR (2020) Stabilization/solidification of strontium using magnesium silicate hydrate cement. Processes 8:163. 10.3390/pr8020163

[60] Zhang T, Fu H, Han J (2023) Deformation mechanisms of magnesium silicate hydrate cement with a shrinkage-reducing admixture under different curing conditions. Minerals 13:563. 10.3390/min13040563

[61] Zhang J, Zhang T, Yuan C, Chen X (2024) Influence of MgO chemical activity on the drying shrinkage of the MgO–SiO_2_–H_2_O system. Constr Build Mater 453:139108. 10.1016/j.conbuildmat.2024.139108

[62] Cheli L, Bonini M, Tonelli M (2024) Effect of sodium phosphate and cellulose ethers on MgO/SiO_2_ cements for the 3D printing of forsterite bioceramics. Appl Sci 14:4410. 10.3390/app14114410

[63] Chen S, Wang L, Wu Z, Zhang T (2019) Mechanical properties and reaction products of reactive magnesia and CFB slag/silica fume pastes. Adv Cem Res 31:297–307. 10.1680/jadcr.16.00171

[64] Bhagath Singh GVP, Sonat C, Yang EH, Unluer C (2020) Performance of MgO and MgO–SiO_2_ systems containing seeds under different curing conditions. Cem Concr Compos 108:103543. 10.1016/j.cemconcomp.2020.103543

[65] Zhang T, Liang X, Li C, Lorin M, Li Y, Vandeperre LJ, Cheeseman CR (2016) Control of drying shrinkage in magnesium silicate hydrate (M-S-H) gel mortars. Cem Concr Res 88:36–42. 10.1016/j.cemconres.2016.05.011

[66] Zhang T, Zhou Z, Li M, He Z, Jia Y, Cheeseman CR, Shi C (2022) Effect of hydrated magnesium carbonate grown &lt;it&gt;in situ&lt;/it&gt; on the property of MgO-activated reactive SiO_2_ mortars. J Sustain Cem Based Mater 11:286–296. 10.1080/21650373.2021.1951880

[67] Sha S, Moghul S, Flatt RJ (2026) New insights into the role of superplasticizer addition time on yield stress of cementitious materials. Cem Concr Res 200:108089. 10.1016/j.cemconres.2025.108089

[68] Quiatchon PRJ, Qiu J (2024) The setting and early-age volume change of hydrated reactive magnesia cement. In: The 12th ConCreep Conference (ConCreep12). Delft, Netherlands

[69] Flatt R, Houst Y, Bowen P, Hofmann H, Widmer J, Sulser U, Mäder U, Bürge T (1997) Interaction of superplasticizers with model powders in a highly alkaline medium. In: 5th Canmet/ACI international conference on superplasticizers and other chemical admixtures in concrete. Detroit, USA

[70] Kauppi A, Andersson KM, Bergström L (2005) Probing the effect of superplasticizer adsorption on the surface forces using the colloidal probe AFM technique. Cem Concr Res 35:133–140. 10.1016/j.cemconres.2004.07.008

[71] Kjeldsen AM, Flatt RJ, Bergström L (2006) Relating the molecular structure of comb-type superplasticizers to the compression rheology of MgO suspensions. Cem Concr Res 36:1231–1239. 10.1016/j.cemconres.2006.03.019

[72] Ferrari L, Kaufmann J, Winnefeld F, Plank J (2010) Interaction of cement model systems with superplasticizers investigated by atomic force microscopy, zeta potential, and adsorption measurements. J Colloid Interface Sci 347:15–24. 10.1016/j.jcis.2010.03.005 20356605

[73] Ferrari L, Kaufmann J, Winnefeld F, Plank J (2011) Multi-method approach to study influence of superplasticizers on cement suspensions. Cem Concr Res 41:1058–1066. 10.1016/j.cemconres.2011.06.010

[74] Tayebi M, Kamala Ilango N, Nguyen H, Lori AR, Ranjbar N, Carvelli V, Kinnunen P (2025) New insights into hydration of MgO in the presence of polycarboxylate ether superplasticizers. Cem Concr Compos 162:106153. 10.1016/j.cemconcomp.2025.106153

[75] Siddique R (2011) Utilization of silica fume in concrete: review of hardened properties. Resour Conserv Recycl 55:923–932. 10.1016/j.resconrec.2011.06.012

[76] Fang Y, Wang X, Jia L, Liu C, Zhao Z, Chen C, Zhang Y (2022) Synergistic effect of polycarboxylate superplasticizer and silica fume on early properties of early high strength grouting material for semi-flexible pavement. Constr Build Mater 319:126065. 10.1016/j.conbuildmat.2021.126065

[77] Wang M, Yao H (2021) Effects of polycarboxylate ether grafted silica fume on flowability, rheological behavior and mechanical properties of cement-silica fume paste with low water-binder ratio. Constr Build Mater 272:121946. 10.1016/j.conbuildmat.2020.121946

[78] Hommer H (2009) Interaction of polycarboxylate ether with silica fume. J Eur Ceram Soc 29:1847–1853. 10.1016/j.jeurceramsoc.2008.12.017

[79] Shah V, Dhakal M, Scott A (2022) Long-term performance of MgO–SiO_2_ binder. Mater Struct 55:60. 10.1617/s11527-022-01901-3

[80] Shah V, Dhandapani Y, Scott A (2021) Pore structure characteristics of MgO–SiO_2_ binder. J Am Ceram Soc 104:6002–6014. 10.1111/jace.17971

[81] Shah V, Scott A (2021) Hydration and microstructural characteristics of MgO in the presence of metakaolin and silica fume. Cem Concr Compos 121:104068. 10.1016/j.cemconcomp.2021.104068

[82] Shah V, Scott A (2021) Assessment of hardened concrete properties of MgO-SiO_2_ binder systems. ACI Mater J 118:223–232. 10.14359/51730418

[83] Dewitte C, Bertron A, Neji M, Lacarrière L, Dauzères A (2022) Chemical and microstructural properties of designed cohesive M-S-H pastes. Materials (Basel) 15:547. 10.3390/ma15020547 PMC8782006 35057269

[84] Zhu Y, German A, Wyrzykowski M, Toropovs N, Winnefeld F, Lura P, Griffa M (2025) Low-carbon MgO/hydromagnesite binders—effect of moisture state on the evolution of mechanical properties. Cem Concr Res 197:107966. 10.1016/j.cemconres.2025.107966

[85] Antoni HJG, Kusuma OC, Hardjito D (2017) Optimizing polycarboxylate based superplasticizer dosage with different cement type. Procedia Eng 171:752–759. 10.1016/j.proeng.2017.01.442

[86] Hay R, Khalil A, Otchere C, Alanis S, Celik K (2023) Proportioning, carbonation, performance assessment, and application of reactive magnesium oxide cement-based composites with superplasticizers. J Mater Civ Eng 35:04022405. 10.1061/(ASCE)MT.1943-5533.0004552

[87] Hänchen M, Prigiobbe V, Baciocchi R, Mazzotti M (2008) Precipitation in the Mg-carbonate system—effects of temperature and CO_2_ pressure. Chem Eng Sci 63:1012. 10.1016/j.ces.2007.09.052

[88] Xu J, Yan C, Zhang F, Konishi H, Xu H, Teng HH (2013) Testing the cation-hydration effect on the crystallization of Ca–Mg–CO_3_ systems. Proc Natl Acad Sci USA 110:17750–17755. 10.1073/pnas.1307612110 PMC3816432 24127571

[89] Bernard E, Lothenbach B, Rentsch D, German A, Winnefeld F (2022) Effect of carbonates on the formation of magnesium silicate hydrates. Mater Struct 55:183. 10.1617/s11527-022-02018-3

[90] Zhao H, Hanein T, Kinoshita H (2023) Effect of sodium bicarbonate (NaHCO_3_) on the development of magnesium silicate hydrate (M-S-H) cement*.* Preprint SSRN. 10.2139/ssrn.4500612

[91] Zhao H, Hanein T, Li N, Alotaibi A, Li A, Walling S, Kinoshita H (2019) Acceleration of M-S-H gel formation through the addition of alkali carbonates. In: 15th Int. Congr. Chem. Cem. (ICCC 2019). Prague, Czech Repuplic

[92] Nied D, Enemark-Rasmussen K, L’Hopital E, Skibsted J, Lothenbach B (2016) Properties of magnesium silicate hydrates (M-S-H). Cem Concr Res 79:323–332. 10.1016/j.cemconres.2015.10.003

[93] Kulik DA, Wagner T, Dmytrieva SV, Kosakowski G, Hingerl FF, Chudnenko KV, Berner UR (2013) GEM-Selektor geochemical modeling package: revised algorithm and GEMS3K numerical kernel for coupled simulation codes. Comput Geosci (Bassum) 17:1–24. 10.1007/s10596-012-9310-6

[94] Wagner T, Kulik DA, Hingerl FF, Dmytrieva SV (2012) GEM-SELEKTOR geochemical modeling package: TSolMod library and data interface for multicomponent phase models. Can Mineral 50:1173–1195. 10.3749/canmin.50.5.1173

[95] Hummel W, Thoenen T (2023) The PSI Chemical Thermodynamic Database 2020. Nagra NTB 21-03

[96] Lothenbach B, Kulik DA, Matschei T, Balonis M, Baquerizo L, Dilnesa B, Miron GD, Myers RJ (2019) Cemdata18: a chemical thermodynamic database for hydrated Portland cements and alkali-activated materials. Cem Concr Res 115:472–506. 10.1016/j.cemconres.2018.04.018

[97] Kuhn R, Lothenbach B, Lura P, Habert G, Bernard E (2026) Effects of carbonate and phosphate admixtures on MgO dissolution and Mg(OH)2 precipitation. Cem Concr Res 204:108207. 10.1016/j.cemconres.2026.108207

[98] Zhang Y, Li Y, Dai Y, Liu J, Xu Y (2018) Hydration evolution of MgO-SiO_2_ slurries in the presence of sodium metasilicate. Ceram Int 44:6626–6633. 10.1016/j.ceramint.2018.01.070

[99] Zhou Z, Bernard E, Lothenbach B (2025) Effect of nesquehonite and silica on magnesia-silicate-carbonate cements. Cem Concr Res 194:107892. 10.1016/j.cemconres.2025.107892

[100] Toroz D, Song F, Uddin A, Chass GA, Di Tommaso D (2022) A database of solution additives promoting Mg^2+^dehydration and the onset of MgCO_3_nucleation. Cryst Growth Des 22:3080–3089. 10.1021/acs.cgd.1c01525 PMC9073943 35529066

[101] Zhang X, Zeng Y, Li Z (2021) Accelerated phase transition and synthesis of magnesite from hydromagnesite in aqueous mono-ethylene glycol solution. J Cryst Growth 563:126105. 10.1016/j.jcrysgro.2021.126105

[102] Dung NT, Unluer C (2017) Sequestration of CO_2_ in reactive MgO cement-based mixes with enhanced hydration mechanisms. Constr Build Mater 143:71–82. 10.1016/j.conbuildmat.2017.03.038

[103] Kamala Ilango N, Nguyen H, German A, Winnefeld F, Kinnunen P (2024) Role of magnesium acetate in hydration and carbonation of magnesium oxide-based cements. Cem Concr Res 175:107357. 10.1016/j.cemconres.2023.107357

[104] Dung NT, Unluer C (2016) Improving the performance of reactive MgO cement-based concrete mixes. Constr Build Mater 126:747–758. 10.1016/j.conbuildmat.2016.09.090

[105] Caulfield B, Abraham J, Christodoulatos C, Prigiobbe V (2022) Enhanced precipitation of magnesium carbonates using carbonic anhydrase. Nanoscale 14:13570–13579. 10.1039/D2NR03199J 36074719

[106] Power IM, Harrison AL, Dipple GM, Southam G (2013) Carbon sequestration via carbonic anhydrase facilitated magnesium carbonate precipitation. Int J Greenh Gas Control 16:145–155. 10.1016/j.ijggc.2013.03.011

[107] Power IM, Harrison AL, Dipple GM (2016) Accelerating mineral carbonation using carbonic anhydrase. Environ Sci Technol 50:2610–2618. 10.1021/acs.est.5b04779 26829491

[108] Hoang T, Dung NT, Unluer C, Chu J (2021) Use of microbial carbonation process to enable self-carbonation of reactive MgO cement mixes. Cem Concr Res 143:106391. 10.1016/j.cemconres.2021.106391

[109] Dung NT, Unluer C (2017) Carbonated MgO concrete with improved performance: the influence of temperature and hydration agent on hydration, carbonation and strength gain. Cem Concr Compos 82:152–164. 10.1016/j.cemconcomp.2017.06.006

[110] Xiao X, Goh LX, Unluer C, Yang E-H (2021) Bacteria-induced internal carbonation of reactive magnesia cement. Constr Build Mater 267:121748. 10.1016/j.conbuildmat.2020.121748

[111] Bernard E (2022) Research progress on magnesium silicate hydrate phases and future opportunities. RILEM Tech Lett 7:47–57. 10.21809/rilemtechlett.2022.162

[112] Bastian A, Emminger YH, Kerdieh N, Bernard E, Ruiz-Agudo C (2025) Insights into the impact of small anionic additives on Mg-silicate hydrate nucleation. J Chem Phys 162:064704. 10.1063/5.0245620 39936516

[113] Marsiske MR, Debus C, Di Lorenzo F, Bernard E, Churakov SV, Ruiz-Agudo C (2021) Immobilization of (aqueous) cations in low pH M-S-H cement. Appl Sci 11:2968. 10.3390/app11072968

[114] Amaral LF, Oliveira IR, Bonadia P, Salomão R, Pandolfelli VC (2011) Chelants to inhibit magnesia (MgO) hydration. Ceram Int 37:1537–1542. 10.1016/j.ceramint.2011.01.030

[115] Bouchaud-Deliot C, Winnefeld F, Lothenbach B (2026) Effect of sodium orthophosphate on rheology and hydration of MgO/nesquehonite cement. J Adv Concr Technol 24:369–381. 10.3151/jact.24.369

[116] Salomao R, Pandolfelli VC (2011) Citric acid as anti-hydration additive for magnesia containing refractory castables. Ceram Int 37:1839–1842. 10.1016/j.ceramint.2011.03.050

[117] Chen L, Jiang LH, Chu HQ, Xu P, Jin WZ, Chen C, Zhi FF, Ben XQ (2020) Effect of retarder on hydration properties of light-burned magnesia. Constr Build Mater 263:119762. 10.1016/j.conbuildmat.2020.119762

[118] Chen L, Jiang LH, Jin WZ, Guo MZ, Niu YL, Chu HQ, Song ZJ (2022) Effect of citrate retarders on the expansion property and hydration behavior of light-burned magnesia cement-based materials. J Build Eng 61:105265. 10.1016/j.jobe.2022.105265

[119] Ma S, Li W, Zhang S, Ge D, Yu J, Shen X (2015) Influence of sodium gluconate on the performance and hydration of Portland cement. Constr Build Mater 91:138–144. 10.1016/j.conbuildmat.2015.05.068

[120] Ruan S, Unluer C (2017) Effect of air entrainment on the performance of reactive MgO and PC mixes. Constr Build Mater 142:221–232. 10.1016/j.conbuildmat.2017.03.068

[121] Li X, Fu Q, Lv Y, Leng D, Jiang D, He C, Wu K, Dan J (2022) Influence of curing conditions on hydration of magnesium silicate hydrate cement. Constr Build Mater 361:129648. 10.1016/j.conbuildmat.2022.129648

